# Data-Driven Blood Glucose Pattern Classification and Anomalies Detection: Machine-Learning Applications in Type 1 Diabetes

**DOI:** 10.2196/11030

**Published:** 2019-05-01

**Authors:** Ashenafi Zebene Woldaregay, Eirik Årsand, Taxiarchis Botsis, David Albers, Lena Mamykina, Gunnar Hartvigsen

**Affiliations:** 1 Department of Computer Science University of Tromsø – The Arctic University of Norway Tromsø Norway; 2 Norwegian Centre for E-health Research University Hospital of North Norway Tromsø Norway; 3 The Sidney Kimmel Comprehensive Cancer Center Johns Hopkins University School of Medicine Baltimore, MD United States; 4 Department of Biomedical Informatics Columbia University New York, NY United States

**Keywords:** type 1 diabetes, blood glucose dynamics, anomalies detection, machine learning

## Abstract

**Background:**

Diabetes mellitus is a chronic metabolic disorder that results in abnormal blood glucose (BG) regulations. The BG level is preferably maintained close to normality through self-management practices, which involves actively tracking BG levels and taking proper actions including adjusting diet and insulin medications. BG anomalies could be defined as any undesirable reading because of either a precisely known reason (normal cause variation) or an unknown reason (special cause variation) to the patient. Recently, machine-learning applications have been widely introduced within diabetes research in general and BG anomaly detection in particular. However, irrespective of their expanding and increasing popularity, there is a lack of up-to-date reviews that materialize the current trends in modeling options and strategies for BG anomaly classification and detection in people with diabetes.

**Objective:**

This review aimed to identify, assess, and analyze the state-of-the-art machine-learning strategies and their hybrid systems focusing on BG anomaly classification and detection including glycemic variability (GV), hyperglycemia, and hypoglycemia in type 1 diabetes within the context of personalized decision support systems and BG alarm events applications, which are important constituents for optimal diabetes self-management.

**Methods:**

A rigorous literature search was conducted between September 1 and October 1, 2017, and October 15 and November 5, 2018, through various Web-based databases. Peer-reviewed journals and articles were considered. Information from the selected literature was extracted based on predefined categories, which were based on previous research and further elaborated through brainstorming.

**Results:**

The initial results were vetted using the title, abstract, and keywords and retrieved 496 papers. After a thorough assessment and screening, 47 articles remained, which were critically analyzed. The interrater agreement was measured using a Cohen kappa test, and disagreements were resolved through discussion. The state-of-the-art classes of machine learning have been developed and tested up to the task and achieved promising performance including artificial neural network, support vector machine, decision tree, genetic algorithm, Gaussian process regression, Bayesian neural network, deep belief network, and others.

**Conclusions:**

Despite the complexity of BG dynamics, there are many attempts to capture hypoglycemia and hyperglycemia incidences and the extent of an individual’s GV using different approaches. Recently, the advancement of diabetes technologies and continuous accumulation of self-collected health data have paved the way for popularity of machine learning in these tasks. According to the review, most of the identified studies used a theoretical threshold, which suffers from inter- and intrapatient variation. Therefore, future studies should consider the difference among patients and also track its temporal change over time. Moreover, studies should also give more emphasis on the types of inputs used and their associated time lag. Generally, we foresee that these developments might encourage researchers to further develop and test these systems on a large-scale basis.

## Introduction

### Background

Diabetes mellitus is a chronic metabolic disorder that results in abnormal blood glucose (BG) regulation. The BG level is maintained close to normality through self-management practices, which involves actively tracking BG levels and taking proper actions including diet and insulin medications. The estimated number of people with diabetes aged between 20 and 79 years was 415 million (uncertainty interval: 340-536 million) in 2015 and is expected to reach 642 million (uncertainty interval: 521-829 million) by 2040 [1]. The global economic burden of diabetes in adults aged between 20 and 79 years was estimated to be US $1.31 trillion (95% CI 1.28-1.36) in 2015 [2]. The total number of deaths attributed to diabetes is estimated to be 5 million in people with diabetes aged between 20 and 79 years [1]. People with diabetes have a higher risk of getting infections as compared with the normal population, which potentially increases their morbidity and mortality [3]. The greater and frequent risk of infections is mainly correlated with a hyperglycemia environment [3,4]. Moreover, studies suggest a hypoglycemia episode could result in a higher hospitalization and mortality rate [5].

The individual’s BG dynamic is affected by various factors, which are mainly categorized as common, individual, and unpredictable factors [6]. The common factors include amount of food intake, insulin intake, previous level of BG, pregnancy, drug and vitamin intake, smoking, and alcohol intake. The individual factors include dawn phenomena, physical exercise load, and menstruation. The unpredictable factors include stress, concomitant diseases, and infections [6]. Swings in BG dynamics, that is, hypoglycemia and hyperglycemia, could be generally categorized under a normal cause variation and special cause variation. The normal cause variation is regarded as caused by those common and individual factors, whereas the special cause variation is caused by those unpredictable factors. The underlying reason of the special cause variations is difficult to understand and remains a challenge for the patient during the incidences. For instance, during stress and infections, the patient usually struggles with hyperglycemia and injects frequent insulin to lower his or her BG levels.

BG anomalies could be defined as any undesirable reading because of either a precisely known reason (normal cause variation) or an unknown reason (special cause variation) to the patient [7]. Even if the advancement in self-management applications and diabetes monitoring technologies has made things easier, the challenge of BG anomalies remains to be managed by the patient themselves. There are some technological developments in the direction of personalized decision systems and BG event alarms to provide an alert and decision support to the patient in the time of these challenges. Techniques such as classification and detection of glycemic variability (GV), hypoglycemia, and hyperglycemia, in particular, and BG anomalies, in general, are central to the development of these diabetes technologies. The ubiquitous nature and widespread use of mobile health (mHealth) apps, sensors and wearables, and other point-of-care (POC) devices for self-monitoring and management purposes have made possible the generation of automated and continuous diabetes-related data, which brought an opportunity for the introduction of machine learning and its application for intelligent and improved systems, which is capable of solving complex tasks within a dynamic knowledge and dynamic environment. In this regard, there are some reviews conducted toward the applications of artificial intelligence in diabetes-related tasks. For instance, Contreras et al [8] conducted literature reviews on the applications of artificial intelligence in the context of critical diabetes management issues such as BG prediction and strategies for BG control, adverse glycemic events detection, bolus calculators and advisory system, patient personalization (tailored features), and others [8]. Moreover, Rigla et al [9] also conducted a review to provide a general overview and popularity of artificial intelligence applications to diabetes problems. Generally, both Contreras et al [8] and Rigla et al [9] tried to demonstrate the potential of artificial intelligence with regard to all groups of people with diabetes focusing on general self-management issues. As far as our knowledge is concerned, there are almost no reviews conducted toward techniques of BG anomaly classification and detection focusing on various approaches, in general, and machine-learning applications, in particular. However, there were some reviews conducted to evaluate the significant effect of pattern management based on self-monitoring BG (SMBG) with regard to clinical practices [10]. Therefore, we suggest that there is a lack of reviews focusing on BG anomaly classification and detection. The objective of this review was to identify, assess, and analyze the state-of-the-art machine-learning strategies in BG anomaly classification and detection including GV, hyperglycemia, and hypoglycemia in people with type 1 diabetes. Moreover, it has presented the current modeling options of machine-learning applications and their hybrid systems. The review covers machine-learning approaches pertinent to personalized decision support systems and BG alarm events applications in type 1 diabetes.

### Machine Learning Tasks in Type 1 Diabetes

**Figure 1:**
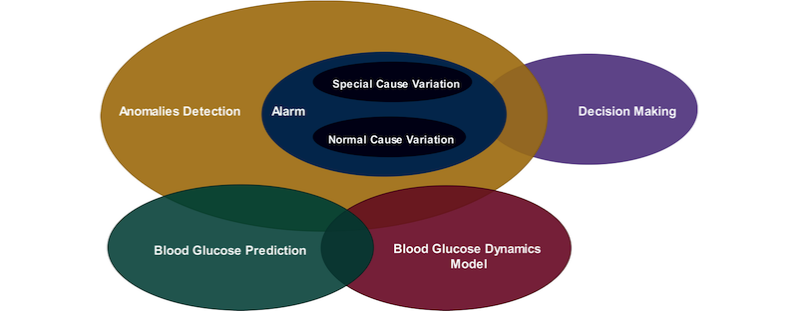
Most widely used machine learning–based data mining tasks based on self-recorded data in people with type 1 diabetes. The yellow shaded ellipse depicts the scope of this review.

Machine-learning approaches (tasks) are generally categorized as regression, prediction, classification, detection, and clustering, which are grouped either in supervised, semisupervised, unsupervised, or reinforcement learning based on the type of learning employed. Generally, reinforcement learning is out of the scope of this review, where we mainly focus on the other 3 categories. Machine learning–based data mining tasks could be categorized as descriptive or unsupervised (ie, clustering, association, and summarization), semisupervised (ie, classification and detection), and predictive or supervised learning (ie, classification and regression) [11]. In this regard, the most widely used machine learning–based data mining tasks in the literature are BG anomalies detection, BG prediction, modeling of BG dynamics, and decision making or education, as shown in Figure 1. In this review, we will focus on the typical applications of classification and detection tasks in diabetes research, specifically in BG anomaly detection within the context of a personalized decision support system and BG alarm events applications. The review considers various classes of machine learning algorithms: artificial neural network (ANN), decision trees (DTs), support vector machine (SVM), evolutionary algorithms (EAs), and others.

An ANN is a biologically inspired computational model consisting of a set of interconnected neurons and a scaled connection between them that is called weights [12]. On the basis of network topology, an ANN is mainly categorized as a feedforward ANN (single-layer perceptron (SLP), multi-layer perceptron (MLP), and radial basis function [RBF]) and feedback ANN (recurrent neural network [RNN], Elman net, Kohonen’s self-organizing map (SOM), and Hopfield networks) [12]. The SVM works based on the theory of structural risk minimization principle [13]. Learning in the SVM occurs through finding an optimal hyperplane that can maximize the margin between the classes. The SVM has been widely exploited in numerous applications such as regression and prediction, pattern identification and recognition, categorization, and classification [13]. An EA is a biologically inspired approach to problem solving [14]. The 2 most used variants of EA are genetic programming (GP) and genetic algorithm (GA). Random forest (RF) or DTs are a kind of an ensemble approach of learning for different classification and regression applications, which mainly learns by constructing a multitude of DTs generating the mode of the class or mean of prediction. The hidden Markov model (HMM) is a variant of the statistical Markov model, where the system being modeled is assumed to follow a Markov property with unobserved states [15]. There are various versions of HMMs; however, in this review, we considered only those trained with a framework close to machine learning families. Hybridization is the process of combining 2 or more different approaches in parallel or serious connection, either at the preprocessing stage, feature extraction, or learning stage, when looking for an improved performance [16].

#### Blood Glucose Anomaly Classification and Detection

Hawkins defined anomalies as “observations that deviate much from the other observations so as to arouse suspicions that it could be generated by a different process” [7,17,18]. There are terms that are often used interchangeably with anomalies, such as outliers, deviations, exceptions, rare instances, and irregularities. The problem of identifying and capturing anomalies in data can be supervised, semisupervised, and unsupervised tasks [19,20]. These strategies can roughly be categorized as classifier- or model-based (detection) approach. The semisupervised is better when anomalous instances are not easily available, whereas supervised techniques are more suitable when there are sufficient labeled instances of both normal and anomalous instances. The unsupervised approach does not require any reference data labels, where normal behaviors have to be determined dynamically, and the detections are mainly performed with regard to the entire datasets. The model-based strategies can be considered as a diagnosis of the system’s behavior during abnormal situations through modeling and adequately characterizing the system’s behavior during normal situations [19,21]. It uses a system’s model to either estimate or predict the underlying system (process) dynamics to capture anomalies in the data. The most important design requirement in using a model includes discovering and characterizing what is to be considered a normal pattern of behaviors [22]. Unlike the classifier-based strategies, the model-based strategies do not require rigorous knowledge of the underlying expected anomalies, that is, to fully understand and characterize the shape and nature of the expected anomalies [22]. By simply defining what is the expected normal pattern the system should exhibit, the model-based anomaly detection is capable of detecting abnormal behavior, which is not considered as the normal behavior of the system. Defining and discovering what is *normal* is a challenging task especially for dynamic and complex systems, for example, BG dynamics. However, this is often tackled in a dynamic and complex system by relying on either a machine learning model trained on a large enough dataset or using an explicit mathematical model, for example, physiological model of BG dynamics, of the system if it exists already.

BG readings are time series data, and anomalies in BG levels could be regarded as any undesirable readings, as shown in Figure 1, because of either a predictable cause (normal cause variation) or an unpredictable cause (special cause variation). A normal cause variation could be defined as any hypoglycemia or hyperglycemia incidences with the underlying cause known to the patient herself or himself and also referred as predictable (patient controllable) factors such as insulin injection, diet intake, physical activity, and others. However, special cause variation refers to any hypoglycemia or hyperglycemia incidences with the underlying cause unknown to the patient and also called unpredictable (patient uncontrollable) factors such as stress, infections, insulin set failure, and others. The classifier, semisupervised (model)– and unsupervised-based approach could be used to solve the challenge of capturing BG anomalies caused by both the predictable factors (normal cause variation) and unpredictable cause (special cause variation). However, regarding the unpredictable factors (special cause variation), the classifier-based approach remains to be very challenging with limited feasibility as the classifier-based strategies require a thorough understanding and characterization of the nature, size, and shape of the anomalies, along with its inter- and intravariability among the patients. With the same token, the unsupervised approach could face the same challenge as it does not have any mechanisms for differentiating the one with special cause from the normal cause variations. However, the model-based (semisupervised) approach happens to be more appropriate given that it only requires to characterize what is considered to be normal so as to detect what is believed to be abnormal. For example, infection (stress)–related hyperglycemia and a diet-induced hyperglycemia are treated differently according to the model-based (semisupervised) anomaly detection strategies. In this regard, diet-induced hyperglycemia is treated as normal, as the model could describe the underlying cause (certain meal), but infection-related hyperglycemia is considered as an anomaly because the model cannot describe the underlying cause based on patient controllable variables (eg, meals and insulin).

GV measures the degree or the rate at which the patient’s BG fluctuates between high and low levels [23]. GV is useful to provide all-inclusive information on one’s self-management practices concerning postprandial spikes in BG, as well as episodes of hypoglycemic and hyperglycemic events [23,24], which are the main factors that contribute for a higher risk of cardiovascular events in people with diabetes. The evaluation of GV helps to comprehend and assess the effect of the patient’s timely actions on the hypoglycemia and hyperglycemia incidence by associating out-of-target BG levels with patient-specific factors, such as insulin dosage, other medication, meals, activity, stress, and illness [23]. However, there is no gold standard approach for assessing GV, and despite its importance, it remains to be challenging.

#### Blood Glucose Prediction

BG prediction is about forecasting an individual’s future BG levels using current and past information and is also an important constituent of BG anomaly classification and detection approaches. It mainly aims to provide crucial alarms for patients in advance with sufficient lead time so as to avoid further complications from hypoglycemia or hyperglycemia incidences. According to Oviedo et al [25], BG prediction models could be categorized into 3 main groups: physiological models, data-driven models, and hybrid models [25]. These categories are solely demarcated based on the necessity of extensive knowledge of the underlying BG dynamics: black box approach (data-driven model), intermediate knowledge (hybrid model), and extensive knowledge (physiological model). The data-driven model, which is mainly referred to as black box model, uses the patient’s continuous glucose monitoring (CGM), insulin, dietary, and other relevant information to develop a prediction model, for example, machine learning and time series approaches. There are a variety of data-driven models developed and tested in the literature including machine learning (neural network, support vector regression, jump neural network, RNN, and others) and time series models (autoregressive [AR] with exogenous input, AR moving average with exogenous input, AR moving average, and others) [25]. Hybrid models make use of the advantages from the data-driven and physiological models [25]. Most of the hybrid models rely on the physiological model to compute meal and insulin information as input for the data-driven models [25]. Physiological models mainly rely on 3 sets of mathematical (differential) equations to describe the underlying dynamics: BG dynamics, insulin dynamics, and meal absorption dynamics. Physiological models are roughly grouped into lumped and comprehensive models based on the way the model treats each organ and tissue so as to develop the differential equations [26]. There are a variety of physiological models developed in the literature such as Berger, Hovorka, Cobelli, Lehmann and Deutsch model, and others [26]. Generally, there are plenty of models implemented in the literature on the prediction of BG levels [25,26]. However, BG prediction is not under the scope of this review, and we mainly focus on the data-driven BG pattern classification and anomaly detection approaches under the umbrella of machine learning.

## Methods

### Search Strategy

The objective of this review was to identify, assess, and analyze the state-of-the-art machine learning strategies and their hybrid system focusing on BG anomaly classification and detection including GV, hyperglycemia, and hypoglycemia in people with type 1 diabetes. The review covers machine learning approaches pertinent to personalized decision support systems and BG alarm events applications. Therefore, for the purpose of the study, a rigorous literature search was conducted between September 1 and October 1, 2017, through various Web-based databases including Google scholar, IEEE Xplore, DBLP Computer Science Bibliography, ScienceDirect, PubMed or Medline, Journal of Diabetes Science and Technology, and Diabetes Technology & Therapeutics. Additional search was also conducted between October 15 and November 5, 2018, on those databases to refine and update the records. Furthermore, the reference list of the selected articles was used to extract additional articles to get a complete overview of the field. Peer-reviewed journals and articles published between 2000 and 2018 were considered. The inclusion and exclusion criteria were setup through rigorous discussion and brainstorming among the authors. Different combinations of terms such as *diabetes*, *intelligent system*, *hybrid system*, *machine learning*, *BG event indicators (hypo- and hyperglycemia prediction)*, *BG event alarm*, *BG personalized decision system*, *clinical*, *closed-loop system*, *hyperglycemia*, *hypoglycemia*, *GV*, and *personalized profile* were used during the search. The terms were combined using *AND/OR* for a better search strategy. Relevant articles were first identified by reviewing the title, keywords, and abstracts for a preliminary filter with our selection criteria, and then we reviewed full text articles that seemed relevant. Information from the selected literature was extracted based on some predefined categories, which were based on previous research, and further elaborated through brainstorming.

### Inclusion and Exclusion Criteria

To be included in the review, the studies should have developed, implemented, tested, and discussed machine learning and any of its hybrid approaches in type 1 diabetes focusing on one or more of the following application areas:

- BG anomaly detection
- Hypoglycemia prediction, classification, or detection
- Hyperglycemia prediction, classification, or detection
- Glycemic or BG variability classification or detection

Therefore, the studies that reside outside of these stated scopes were excluded from the review including all articles written in other languages but English.

### Data Categorization and Data Collection

Information was extracted from the selected studies based on predefined parameters (variables) and categories. The categories were defined based on rigorous brainstorming and discussion among the authors. These categories were demarcated solely to collect the relevant data and to assess, analyze, and evaluate the model’s characteristics and its experimental setup.

#### Application Scenario

This category defines the type of applications where the machine learning algorithm is being exploited. It can be hypoglycemia and hyperglycemia prediction, classification and detection, or GV classification and detection.

#### Type of Input

This category was defined to assess, analyze, and evaluate the type of inputs used to develop the algorithm. This includes the key diabetes parameters and other physiological parameters relevant for BG anomaly classification and detections: BG, heart rate variability, and others.

#### Data Format, Type, Size, and Data Source

This category was defined to assess, analyze, and evaluate the type of data format used as input to the algorithm. This depends on the basis of the type of diabetes technologies, mobile apps, and POC devices used for data collection and algorithm development. It includes different data formats such as from CGM devices, mHealth apps (ie, diabetes diary), heart rate monitoring devices, and others.

#### Input Preprocessing

This category defines the kind of preprocessing algorithm the system implements so as to avoid missing, sparse, and corrupted input data.

#### Class of Machine Learning

This category defines the class of machine learning algorithm used to train and test the BG anomaly classification and detection algorithm. It includes different classes of machine learning algorithm: ANN, SVM, Bayesian network, DT, and others.

#### Training or Learning Method and Algorithm

This category defines the class of learning algorithms used to train the model. It includes different training algorithms such as the backpropagation algorithm, kernel, optimization techniques, and others.

#### Performance Metrics or Evaluation Criteria

This category defines the type of evaluation metrics used to assess the accuracy of the classification and detection algorithm implemented. It includes different performance metrics such as specificity, sensitivity, receiver operating characteristic (ROC) curves, and others.

### Literature Evaluation

The included literature was analyzed and evaluated based on the above defined categories and variables to uncover the state-of-the-art machine learning applications in hyperglycemia or hypoglycemia prediction, classification and detection, and GV classification and detection. It also tries to pinpoint their characteristics along with the experimental setup used to implement and test the algorithms. The first evaluation and analysis was carried out based on the type of input used to develop the algorithms to uncover the state-of-the-art inputs used in these circumstances. The second evaluation and analysis was carried out based on the various classes of machine learning used to develop these algorithms to uncover the rate of adoption and their suitability to the task. The third evaluation and analysis was carried out based on the performance metrics used to evaluate the performance of these algorithms.

## Results

### Relevant Literature

**Figure 2:**
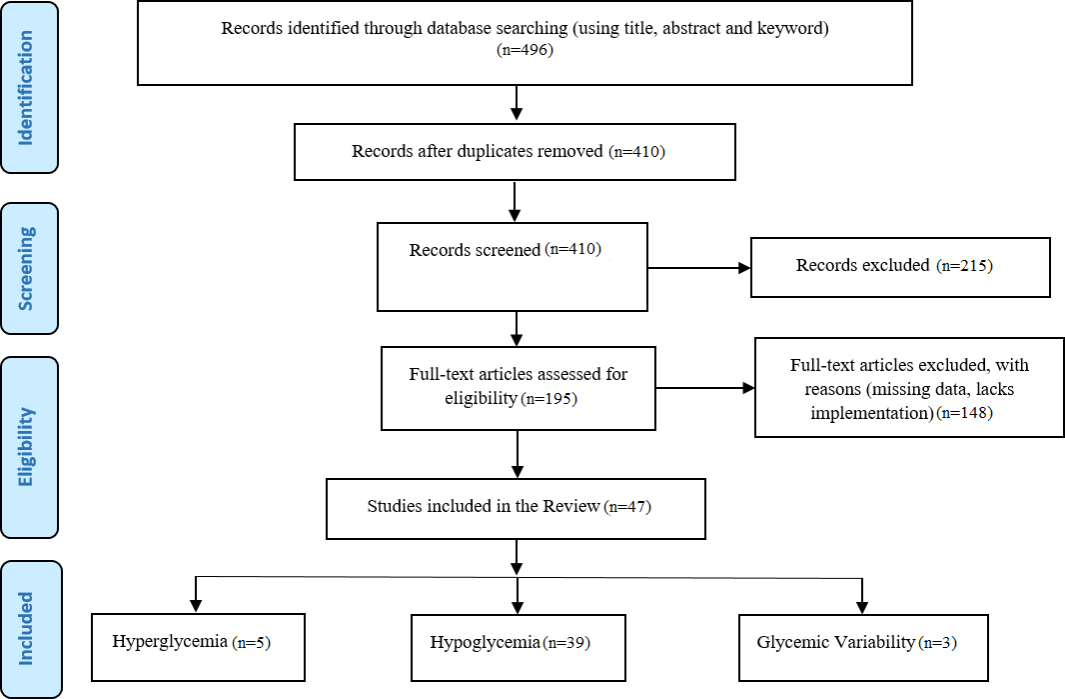
Flow diagram of the review process.

**Figure 3:**
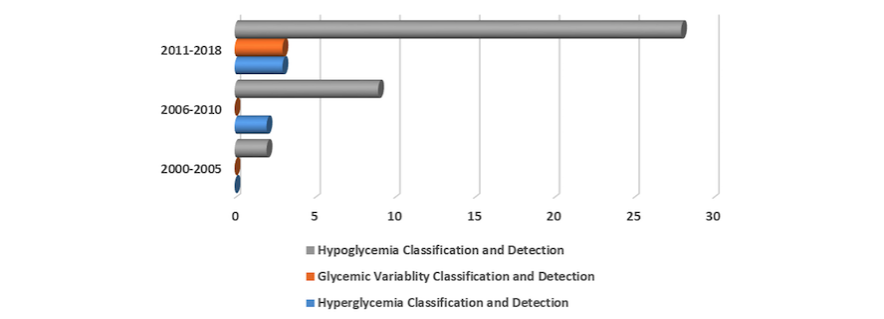
The number of articles published per year of publication.

The initial hit was vetted using the title, abstract, and keywords and retrieved a total of 496 papers (DBLP Computer Science (20), Diabetes Technology & Therapeutics (23), Google Scholar (160), IEEE (215), Journal of Diabetes Science and Technology (22), PubMed Medlin (27), and ScienceDirect (29); see Figure 2). After removing duplicates from the list, 410 records remained. Then, we did an independent assessment of the articles and screening based on the inclusion and exclusion criteria, which eliminated another 215 papers, leaving 195 relevant papers. After a full-text assessment, 47 articles were left (hyperglycemia=5, glycemic variabilities=3, and hypoglycemia=39), which were critically analyzed as shown in Figures 2 and 3. The interrater agreement was measured using a Cohen kappa test, and disagreements were resolved through discussion.

### Evaluation and Analysis of the Literature

The literature, as described previously, was evaluated based on the type of machine learning used to develop the algorithm, the type of input used to train the system, and the performance metrics used to evaluate the algorithm performance based on the tables in Multimedia Appendices 1 and 2.

#### Data Characteristics and Input Parameters

##### Input Parameters

**Figure 4:**
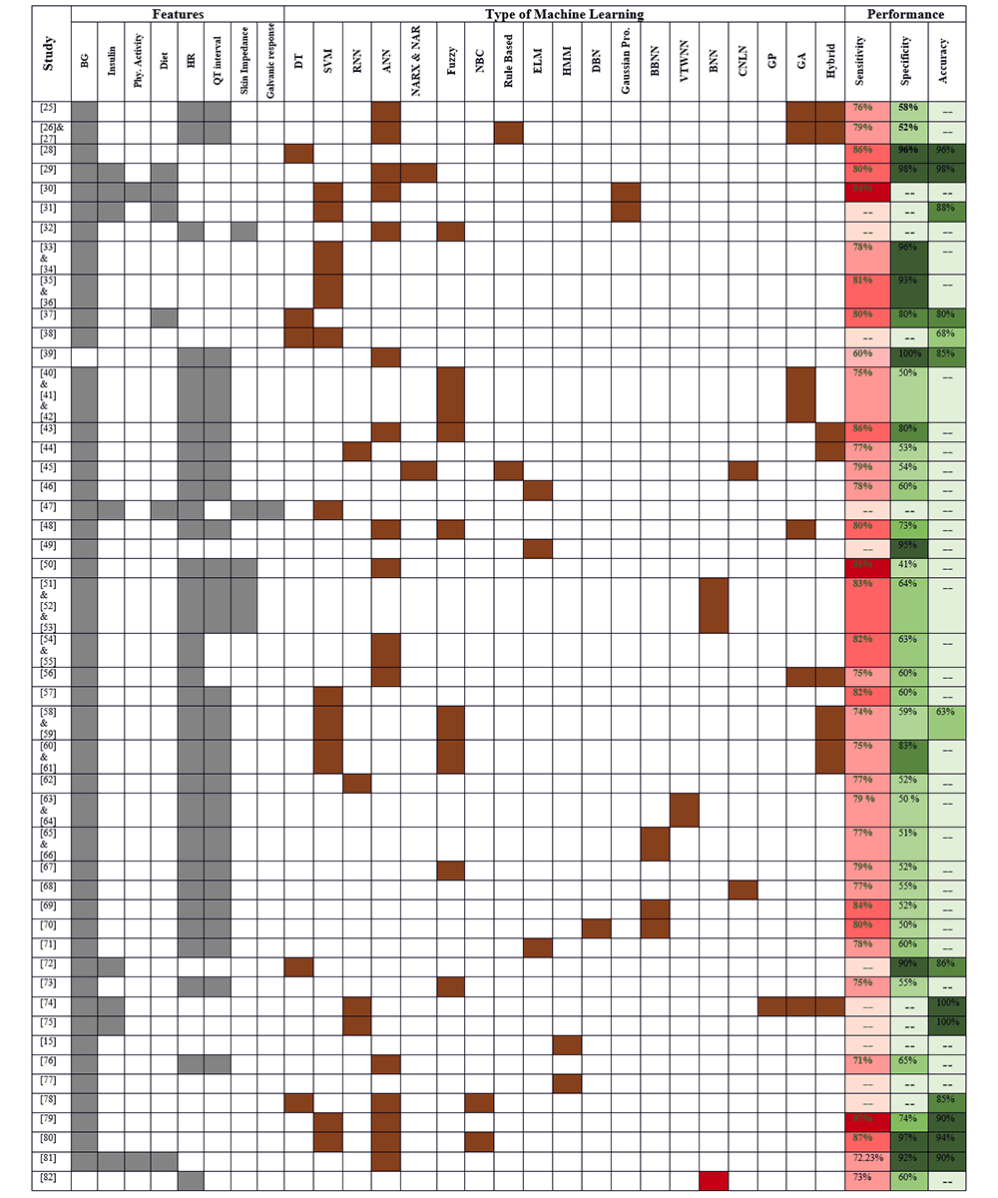
Reported input features, machine learning class, and accuracy. ANN: artificial neural network; BBNN: block-based neural network; BG: blood glucose; BNN: Bayesian Neural Network; DBN: deep belief network; DT: decision tree; ELM: extreme learning machine; GA: genetic algorithm; GP: genetic programming; HMM: hidden Markov model; NAR: nonlinear autoregressive network; NARX: nonlinear autoregressive network with exogenous inputs; NBC: Naive Bayes classifier; RNN: recurrent neural network; SVM: support vector machine; VTWNN: variable translation wavelet neural network.

Selecting the proper types of input parameters is one of the crucial design strategies for successful classification and detection algorithm development. In this regard, the outer bigger ring, the middle ring, and the inner ring in Multimedia Appendix 1 depict the types of input used in hypoglycemia, hyperglycemia, and GV classification and detection algorithm, respectively. According to hypoglycemia classification and detection algorithm, BG, heart rate, and QT interval are the most used types of input parameters (25/39, 64%). BG alone is the second most used type of input parameter (4/39, 10%). BG and insulin are the third most used types of input parameters along with BG, insulin, diet, physical activity, and others (3/39, 8%). BG and diet alone, along with BG, insulin, and diet, and BG, heart rate, skin impedance, and BG, insulin, diet, heart rate, galvanic response, skin impedance are the fifth most used types of input parameters (1/39, 3%). According to hyperglycemia classification and detection algorithm, BG alone, and BG and insulin represent the most used types of input parameters (2/5, 40%). BG, heart rate, and QT interval represent the second most used types of input parameters (1/5, 20%). According to GV classification and detection algorithm, BG alone (3/6, 50%), and BG and insulin (3/6, 50%) are equally ranked as the most used types of input parameters, as shown in Figure 4.

##### Data Characteristics

###### Data Sources

Different kinds of data sources ranging from BG monitors, physical activity, electrocardiogram (ECG), and heart rate sensors have been used in the reviewed articles for hyperglycemia, hypoglycemia, and GV classification and detection algorithms. The reviewed articles relied on different kinds of data formats including SMBG (finger sticks), CGM, and ECG signals, as shown in Table 1. Generally, ECG signal is the most used type of data format (51%), followed by CGM (39%) and SMBG (10%). Specifically, hypoglycemia classification and detection involve (CGM (n=11), ECG (n=24), and SMBG (n=5)). Regarding, hyperglycemia classification and detection (CGM (n=5) and ECG (n=1)) and GV classification and detection (CGM (n=3)).

**Table 1:** Types of data formats used in the studies (N=49).

Data type/format	Count, n (%)
Continuous glucose monitoring	19 (39)
Self-monitoring blood glucose	5 (10)
Electrocardiogram signal	25 (51)

With regard to BG monitoring, different devices and brands have been exploited for developing hypo-/hyperglycemia and GV classification and detection algorithms, as shown in Table 2. Generally, Yellow Spring Instruments is the most used device (50%) followed by Guardian Real Time (MinMed CGM; 28%). GlycoMark (7%) is the third most used device followed by HemoCue Glucose 201 (5%) and Self-Monitored BG (5%). Specifically, the most used devices for hypoglycemia classification and detection are Guardian Real Time (MinMed, CGM; n=7), Yellow Spring Instruments (n=21), HemoCue Glucose 201 (n=2), Dexcom CGM system (n=1), Self-Monitored BG (SMBG; n=2), Medtronic Enlite CGM sensors (n=1), Medtronic insulin pump (n=4), SensorWear armband (physical activity; n=2), and Basis Peak fitness band (n=1). and Basis Peak fitness band (n=1). As for hyperglycemia classification and detection, Guardian Real Time (MinMed CGM; n=2) and Medtronic insulin pump (n=3) had been used. With regard to GV classification and detection, GlycoMark (n=3), Guardian Real Time (MinMed CGM; n=3), and Medtronic insulin pump (n=3) had been used.

**Table 2:** Types of devices used for the monitoring of blood glucose levels (N=42).

Devices	Count, n (%)
Guardian Real Time (MinMed, CGM^a^)	12 (28)
HemoCue Glucose 201 (HemoCue)	2 (5)
Yellow Spring Instruments	21 (50)
Dexcom CGM system	1 (3)
Medtronic Enlite CGM sensors	1 (3)
GlycoMark	3 (7)
Self-Monitored Blood Glucose-unknown device	2 (5)

^a^CGM: continuous glucose monitoring.

Various brands of physiological monitoring (heart rate and ECG signals) devices have been exploited in the reviewed articles. Generally, as shown in Table 3, Compumedics system is the most used system (52%) followed by a customized device such as a battery-powered chest belt–worn device (22%). HypoMon is the third most used device (13%) followed by Basis Peak fitness band (9%) and a self-designed portable apparatus (4%). Specifically, for hypoglycemia classification and detection purposes, various devices have been used such as HypoMon (n=3), Basis Peak (n=2), Compumedics system (n=11), a battery-powered chest belt–worn (n=5), and self-designed portable apparatus (n=1). With regard to hyperglycemia classification and detection, only 1 article has used the Compumedics system (n=1), which indicates that heart rate and ECG signals have a limited use in this case.

**Table 3:** Types of devices used for the monitoring of physiological parameters (heart rate and electrocardiogram signals; N=23).

Devices	Count, n (%)
HypoMon	3 (13)
Basis peak fitness band	2 (9)
Compumedics system	12 (52)
A battery-powered chest belt–worn (customized)	5 (22)
Self-designed portable apparatus (customized)	1 (4)

###### Data Preprocessing

Data preprocessing is an important stage of any machine learning strategy. In this regard, there were various kinds of data preprocessing strategies used in the reviewed articles. The reviewed articles had relied on both BG and other physiological (heart rate, ECG, skin impedance, and others) data, which of course involves different preprocessing strategies depending on the data type under consideration. Regarding the BG data, various preprocessing approaches had been used including differencing (derivative) BG values [27,28], CGM data reconstruction, or smoothing using different methods such as spline interpolation [29-33], a rough feature elimination, such as fast *separability* and correlation analysis algorithm [28,29], representing BG temporal change information [34], feature selection and feature ranking [35], filtering using Pearson’s correlation coefficient (PCC) and the *t* test, and the wrapper approach using greedy backward elimination [33]. The other physiological parameters (heart rate, ECG, skin impedance, and others) had been preprocessed using different methods such as normalization [36-38], feature extraction and selection [39,40], feature extraction using fast Fourier transform (FFT) [41], unsupervised restricted Boltzmann machine–based feature representation [42], filtering techniques such as Infinite impulse response high pass filter [41,43], correlation analysis [44-46], and transformation of frequency domain into time domain (FFT) [47].

#### Class of Machine Learning

##### Hypoglycemia Classification and Detection

Different classes of machine learning techniques have been adopted in hypoglycemia prediction, classification, and detection algorithms to predict, classify, and detect the incoming hypoglycemia incident in people with type 1 diabetes, as shown in Figure 4. Conventional feedforward ANN is the most adopted class of machine learning, which is used in 26% (17/65) of the studies , as shown in Multimedia Appendix 1. Hybridization of machine learning techniques with other approaches such as time series, fuzzy logic, and others are the second most adopted approach (12/65, 18%). The SVM ranked the third most adopted class of machine learning (9/65, 14%). DT ranked the fourth most adopted technique (4/65, 6%). GA, time delay ANN and time sensitive ANN, block-based neural network (BBNN), and adaptive neural fuzzy inference system (ANFIS) are the fifth most used classes of machine learning (3/65, 5%). Nonlinear autoregressive network with exogenous inputs (NARX) and nonlinear autoregressive network (NAR) along with Gaussian process regression, combinational neural logic network , and Bayesian neural network (BNN) ranked as the sixth most used classes of machine learning (2/65, 3%). Deep belief network (DBN), radial basis function neural network (RBFNN), and variable translation wavelet neural network (VTWNN) are the seventh most used classes of machine learning (1/65, 2%).

##### Hyperglycemia Classification and Detection

Hyperglycemia classification, prediction, and detection has been practiced less when compared with hypoglycemia, which might be linked because of its less severe short-term complications as opposed to hypoglycemia incidences. However, irrespective of this limitation, different types of machine learning techniques have been adopted, as shown in Figure 4. For example, ANN is the most used machine learning technique in 34% (3/9) of the studies (feedforward (1/9) and feedback RNN (2/9)), as shown in Multimedia Appendix 1 along with EA (3/9,34%) (GA (1/9) and GP (2/9)). The HMM (2/9, 22%) is the third most used followed by a hybrid approach (1/9, 11%).

##### Glycemic Variability Classification and Detection

GV detection is a recent development, which has great importance in quantifying factors associated with hypo-/hyperglycemia incidence. In this regard, there is some research and development involving machine learning techniques, as shown in Figure 4. For example, feedforward ANN is the most used class of machine learning (3/8, 37%), as shown in Multimedia Appendix 1. Naive Bayes classifier (NBC) and SVM are the second most adopted techniques of machine learning (2/8, 25%). DT is the third most used class of machine learning (1/8, 13%).

#### Performance Metrics

The performance metrics used in the evaluation of hypoglycemia, hyperglycemia, and GV classification and detection algorithms are depicted in the outer ring, the middle ring, and inner ring, respectively, as shown in Multimedia Appendix 1. According to hypoglycemia classification and detection, sensitivity, and specificity are the most used performance metrics (37/58, 64%). Accuracy and precision are the second most used performance metrics (9/58, 15%). Root mean square error and mean square error are the third most used performance metrics (4/58, 7%). Geometric mean is the fourth most used performance metric (3/58, 5%). Correlation coefficient is the fifth most used performance metric (2/58, 3%). Time lag (TL), recall, and ROC curve are the sixth most used performance metrics (1/58, 2%). According to hyperglycemia classification and detection, accuracy and precision, root mean square error and mean square error, time lag (TL), correlation coefficient, recall, and false positive rate are the most used performance metrics (2/15, 13%). ROC curve, geometric mean, sensitivity, and specificity are the third most used performance metrics (1/15, 7%). According to GV classification and detection, accuracy, and precision are the most used performance metrics (3/5, 60%). Sensitivity and specificity are the second most used performance metrics (2/5, 40%).

## Discussion

### Principal Findings

The objective of this review was to identify, assess, and analyze the state-of-the-art machine applications in BG pattern classifications and anomaly detection: hyperglycemia, hypoglycemia, and GV classification and detection. According to the reviewed literature, the anomaly classification and detection approach could be roughly categorized as either a classifier-based or a model-based approach [19,21]. The classifier-based approach mainly relies on using either a specified threshold or some kinds of rules to classify the BG levels as either normal or abnormal. The difference is that unlike the model-based approach, the classifier-based approach requires rigorous and deeper knowledge regarding the nature, size, and shape of the underlying anomalies under consideration so as to develop the necessary threshold or rule to capture them. However, the model-based approach only requires to demarcate the boundary of what is known to be normal so as to capture what is believed to be abnormal [21]. The model-based approach does not require rigorous knowledge of the underlying expected anomalies, that is, to fully understand and characterize the shape and nature of the expected anomalies [22]. By simply defining what is the expected normal pattern that the system should exhibit, a model-based approach is capable of detecting abnormal behavior, which is not considered as the normal behavior of the system. Defining and discovering what is *normal* is a challenging task especially for dynamic and complex systems, for example, BG dynamics. However, this is often tackled in a dynamic and complex system by either relying on a machine learning model trained on large enough datasets or using an explicit mathematical model of the system such as a physiological or compartmental BG dynamics model [21].

Various classes of machine learning algorithms have been adopted for the task. Regarding hypoglycemia classification and detection, feedforward ANN, hybrid systems, SVM, DT, GA, ANFIS, NARX, NAR, Gaussian process regression, DBN, and BNN have been developed and tested. These techniques have explored various kinds of input parameters notably BG, heart rate, QT interval, insulin, diet, physical activity, galvanic response, and skin impedance. Concerning hyperglycemia classification and detection, RNN, GP, HMM, feedforward ANN, GA, and hybrid systems have been developed and tested, exploring various types of input parameters including BG, insulin, heart rate, and QT interval. GV detection is a recent development, which has great importance in quantifying factors associated with hypoglycemia and hyperglycemia incidence. In this regard, there is some research and development involving machine learning techniques. For example, feedforward ANN, NBC, SVM, and DT have been tested up to the task using BG and insulin delivery profiles.

Generally, all of the studies have relied on either indirect indicator variables such as heart rate, QT interval, and others or a subset of input parameters that affects BG dynamics. The patient’s contextual information, for example, meals, physical activity, insulin, and sleep, have a significant effect on BG dynamics, and a proper anomaly classification and detection algorithm should consider the effects of these parameters. In this regard, however, the individual patient is expected to record meal, insulin, and physical activity data. One of the main limitations is meal modeling, where most of the algorithm depends on the individual estimation of carbohydrate, which is prone to errors and further aggravates the degradation to detection performance. With regard to physical activity, there are various wearables and sensors that can record the individual’s physical activity load and durations. However, there is the lack of a uniform approach among the studies with certain limitations on the way these signals are employed in the classification and detection algorithms. For example, there are some studies that consider levels of activity as low, moderate, and high and others consider descriptive features by summarizing the number, intensity, steps, exercise durations, and others to better quantify the effect of physical activities. Moreover, recording insulin dosage has its inherent limitations, which might affect the detection performance. For example, blockage of insulin flow from the insulin pump because of the infusion set failure and error incurred during manual registrations might pose a significant challenge in the performance of the detection system. Furthermore, CGM is becoming one of the most important components in these classification and detection algorithms. However, even if CGM advancement has enabled patients to have continuous estimation of their subcutaneous glucose levels, it has limitations when used in a personalized detection system (an alarm). In this regard, recent studies have showed that autocorrelation of the CGM reading vanishes after 30 min, making the detection performance to degrade afterward. These findings suggest that any classification and detection algorithms aiming for a better lead time should consider other patient’s contextual information and various features of the CGM itself. There are some studies that develop a model by assessing several features of the CGM signal so as to compensate for its inaccuracy. Moreover, CGM is found to be inaccurate during hypoglycemia episodes, that is, insulin-induced hypoglycemia versus spontaneous hypoglycemia. In this regard, insulin-induced hypoglycemia is found to be difficult to detect as compared with spontaneous hypoglycemia. Fast occurring hypoglycemia is difficult to detect because of the blood-interstitial delay, which makes them important features to be detected by a given model. Furthermore, CGM calibration frequency and timing also affects the performance of the classification and detection algorithm.

The reviewed studies are limited to and could be roughly categorized by age groups (children, young, adult, and old), time of the day (diurnal vs nocturnal) and configurations (online vs offline). For example, most of the studies consider nocturnal hypoglycemia detection, considering the fact that most of hypoglycemia crises occurred during nighttime and also the crises during this time have a bad consequence as compared with the diurnal period. Moreover, it is a fact that nocturnal detection is simpler as compared with the diurnal considering the dynamics of the patients. However, irrespective of these challenges, there are also studies that consider the diurnal period. However, there are limited studies that attempt to develop an algorithm that could detect anomalies in both of those contexts. With regard to the age group, most surveys reported that age group has a great effect on BG dynamics, which is typically related with the dynamics and active lifestyle adopted by each group. Therefore, it is deemed a necessary approach to consider a personalized algorithm for each age group. With regard to the configuration, there are fewer attempts of online (real-time) algorithms, where almost all of the algorithms were tested and implemented in the offline mode. In this regard, the most crucial issues concerning machine learning strategies could be the necessity of frequent retraining when subjected to a real-time and dynamic task.

In addition, the most important component in classification and detection algorithms is the threshold used to differentiate the normal from the abnormal. In this regard, almost most of the studies have used a static threshold based on suggestions either from the literature or physicians and other concerned bodies such as the American Diabetes Association. However, the critical issues in this approach are that the threshold might vary from patient to patient and also some patients might not feel any symptoms at the specified threshold (when using indirect indicators such as heart rate, QT interval, and others). However, there are some studies that employed a fuzzy logic–based approach by having a continuous decision space.

In principle, any future BG anomaly classification and detection algorithm should be expected to detect any upcoming anomalies as soon as possible (lead time—giving more response time), avoid any false alarm at any cost, perform in real-time (in an online fashion), adapt with the dynamics of BG evolution (learn continuously), automatically tune its parameters without user intervention, be able to perform throughout the day in a free living condition (diurnal and nocturnal periods), and incorporate as many input variables to better capture the dynamics. In this regard, for example, the most crucial issues concerning a real-time (online) machine learning algorithm could be the necessity of frequent retraining when subject to a real-time and dynamic task. Moreover, developing a model that considers a real-time and adaption-to-free-living condition needs to incorporate a wide range of parameters that affect BG dynamics. Furthermore, it should properly consider and address the inherent technological limitation that affects the performance of the detection algorithm. Almost all of the studies need a proper clinical validation to be integrated into a smartphone and CGM for a real-time application. This can be better described by looking at the number of samples used and their validation strategies (see Multimedia Appendix 2). Therefore, future studies should give more emphasis on clinical validation by taking a sufficient number of subjects in the development and testing phase so as to better quantify the inter- and intravariability among patients. In addition, the most crucial concept of justifying and reporting the underlying cause, as because of either patient controllable or patient uncontrollable parameters, for the detected anomalies is not addressed in any of the reviewed literature. For example, the underlying cause of hyperglycemia incidences could be patient controllable parameters such as diet or patient uncontrollable parameters such as stress and infections. Therefore, in this regard, a proper hyperglycemia classification and detection system might be expected to be able to identify and report the underlying cause, which has a greater significance to the patient especially during infection crises.

### Summary of Existing Efforts: Machine Learning Techniques

#### Artificial Neural Network

There are various types of ANNs used in solving BG classification and anomaly detection tasks: hypoglycemia, hyperglycemia, and GV classification and detection. Regarding hypoglycemia classification and detection, for instance, Eljil et al [48], had proposed a special type of ANN known as the time-sensitive ANN and compared the result with a time delay neural network, NARX, distributed time delay neural network, and NAR. San et al [37,49] proposed an evolvable BBNN and compared the result with feedforward ANNs and multiple regression. Moreover, San et al [42] proposed a DBN and compared the result with a wavelet neural network, a feedforward ANN, and multiple regression models. Some of the studies have also investigated the advantage of having a separate feature extraction and classification unit. In this regard, for example, both Laione et al [47] and Nguyen et al [41,50] have proposed an ANN using FFT for data extraction. Nguyen et al [41] have further trained the network through a 2-step process that combines the advantage of GA and the Levenberg Marquardt algorithm. Chan et al and Yan et al [51,52] also proposed a neural network–based rule discovery system that consisted of a neural network–based classification unit and rule-based extraction unit. There are some studies that optimized the ANN parameters through a particle swarm optimization technique. For example, Ling et al [53], Phyo et al [36,54,55], and San et al [56] proposed a new hybrid rough neural network, a VTWNN, a normalized RBFNN, and a combinational neural logic network with the neural logic AND, OR, and NOT gates, respectively, where the design parameters of the network were optimized through a hybrid particle swarm optimization with wavelet mutation operation. Moreover, Nguyen et al [43,57] also proposed an ANN that is optimized through a standard particle swarm optimization strategy. Furthermore, some studies have investigated extreme learning machines (ELMs). For instance, Ling et al [58] and San et al [59] proposed a feedforward ANN trained through an ELM and compared the result with a feedforward ANN optimized through particle swarm optimization, multiple regression–based fuzzy inference system, fuzzy inference system, and linear multiple regression. Mo et al [60] have also used ELMs and regularized the ELMs on CGM data. In addition, Nguyen et al [61-63] and Ngo et al [64] had proposed an optimal BNN algorithm using feedforward ANN architecture. There are some studies that tried to integrate a physiological model with ANN. For instance, Bertachi et al [65] integrated the physiological model of an individual diabetes patient with an ANN to predict nocturnal hypoglycemia events. Regarding, hyperglycemia classification and detection, there is only 1 study by Nguyen et al [38] that uses a feedforward multilayer ANN trained using different training algorithms, that is, gradient descent, gradient descent with momentum, scaled conjugate gradient, and resilient back propagation. Regarding GV classification and detection, the reviewed studies had been performed either for detection purposes or for automated metrics purposes. For the detection purpose, for example, Wiley et al [33] proposed Naive Bayes (NB), multilayer perceptron (MLP) ANN, and SVM models to detect excessive GV on CGM data and compared the accuracy of the result with the other 2 diabetes experts. Concerning the automated metrics, Marling et al [66] had developed an NBC (probabilistic reasoning), an MLP ANN, and a logistic model tree (DT built using logistic regression), which could be used to monitor CGM data. Moreover, Marling et al [32] also proposed an MLP ANN and support vector regression to develop a consensus perceived GV metric.

#### Support Vector Machines, Kernel Function, and Gaussian Process Regression

SVM, kernel function (KF), and Gaussian process regression have been exploited for hypoglycemia classification and detection purposes in the reviewed literature. For example, Georga et al [67] developed a support vector regression for hypoglycemia prediction and compared the performance with a feedforward MLP ANN and Gaussian process regression. Georga et al [68] also proposed support vector regression and Gaussian process regression for BG prediction so as to indicate the daily incidences of hyperglycemia and hypoglycemia to the patients as well as provision of decision support to physicians in making the decision about treatment and risk of complications. Moreover, Jensen et al [29,30] developed an automatic pattern recognition system so as to detect hypoglycemia incidences retrospectively using CGM data and thereby to foster a thorough evaluation of past events and discussion with their caregivers. Jensen et al [28,69] also proposed a real-time pattern classification model by using several features from the CGM data so as to detect hypoglycemia incidences in real-time. Furthermore, Marling et al [70] proposed a hypoglycemia detection algorithm that incorporates noninvasive sensor data from fitness bands and also compared different kernels for the task: linear, Gaussian, and quadratic kernels. Nuryani et al [71] also proposed a swarm-based SVM algorithm using the repolarization variabilities as input so as to detect hypoglycemia incidences.

#### Genetic Programming and Genetic Algorithm

There is little visibility of GP and GA usage in their nonhybrid form for BG classification and anomaly detection tasks: hypoglycemia, hyperglycemia, and GV classification and detection. However, there are some studies that use these techniques in their hybrid form. For example, Ling et al [44,72,73] developed a hypoglycemia detection algorithm using a GA-based multiple regression coupled with a fuzzy inference system. The study exploited the GA so as to optimize the fuzzy rules, membership function of the fuzzy inference system, and also model parameters of the regression.

#### Random Forest

RF and DT have been mostly used in the context of hypoglycemia classification and detection tasks. For example, Eljil et al [27] proposed DTs using different techniques, namely, C4.5, J4.8, REPTree, bagging, and the cost-sensitive version of J4.8. Jung et al [74] also proposed DTs using new predictor variables using CGM data. Moreover, Jung et al [34] proposed a DT- and SVM-based prediction model using self-monitored BG. Zhang et al [35] also proposed a new approach using the classification tree to predict the occurrences of acute hypoglycemia during intravenous insulin infusion before the actual hypoglycemic events take place.

#### Hidden Markov Model

Generally, HMM is used to model an environment that could better describe the evolution of the individual BG dynamics. In this regard, there are some studies that use HMM to develop model-based BG anomaly classification and detection algorithms. For example, Zhu et al [15,75] studied an approach for automatic detection of anomalies in individual BG data, using a model trained with historical data containing daily normal measurements. The trained Markovian world tries to analyze the incoming BG data and flags if it deviates from what is known by the model.

#### Hybrid and Ensemble Models

Hybridization approaches have been extensively used when looking for performance improvement by exploiting the advantage from 2 or more different approaches [16]. In this regard, there are some attempts in the reviewed articles which tried merging different approaches for enhanced performance in hypoglycemia classification and detection. For example, hybridization of an ANN with other techniques is demonstrated in some of these studies. Chan et al [76] developed a hybrid system that consisted of an ANN and a GA and also compared the performance with MLP ANN and classical statistical algorithms. Ghevondian et al [77] proposed a novel hybrid system of a fuzzy neural network ANN estimator to predict the BG profile and hypoglycemia incidences. San et al [78] proposed a hybrid system using an ANFIS and compared the performance with the wavelet neural network, feedforward ANN, and multiple regression. There is also some literature that tries to hybridize the SVM with other techniques. For example, Nuryani et al [39,79] proposed a hybrid fuzzy SVM and investigated the applicability of 3 KFs: radial basis, exponential radial basis, and polynomial function for the task. Moreover, Nuryani et al [40,80] also further developed a novel strategy using a hybrid particle swarm-based fuzzy SVM technique. Fuzzy reasoning models are also tested in some of the studies. For example, Ling et al [81] developed a hybrid particle swarm-optimization–based fuzzy reasoning model, where the fuzzy rules and the fuzzy-membership functions are optimized through a hybrid particle swarm optimization with wavelet mutation. The model is also compared with feedforward ANN and multiple-regression models. Mathews et al [46] developed a hybrid model using a fuzzy inference system with multiple regression, where the fuzzy rules are optimized through a GA. The study also compares the performance of the developed system with an ANN whose parameters are optimized through particle swarm optimization. In addition, San et al [82] proposed a hybrid system based on rough sets concepts and neural computing. The study has compared various hybrid approaches trained through hybrid particle swarm optimization with wavelet mutation including the rough BBNN, BBNN, rough feedforward ANN, wavelet neural network, SVM with an RBF, and conventional feedforward ANN. Ling et al [45] also proposed an alarm system based on the hybrid neural logic network with multiple regression. Lai et al [83] developed a fuzzy inference system for hypoglycemia detection, where the system parameters are optimized through an intelligent optimizer.

Owing to the complexity of BG dynamics, it remains difficult to achieve an accurate result in every circumstance. One model can have better accuracy in some circumstances and the other model can achieve better accuracy where the first model fails to achieve a comparable result. Therefore, it is natural to look for possibilities to exploit the strengths from these different models to achieve better accuracy in most of the circumstances, which lead to ensemble approaches [16]. An ensemble approach is generally favored when one is interested to merge 2 or more different models for improved performance. In this regard, there are some studies that try to combine 2 different models looking for performance improvement in the overall system. In this regard, Daskalaki et al [84] proposed an early warning system, for both hyperglycemia and hypoglycemia, using RNN and AR with output correction module models. Moreover, the study investigated the performance improvement from the combined use of both RNN and AR with an output correction module. Moreover, Botwey et al [31] proposed combining an AR model with output correction and an RNN based on different data fusion schemes including the Dempster-Shafer evidential theory, GAs, and GP.

### Conclusions

Despite the complexity of BG dynamics, there are many attempts to capture hypoglycemia and hyperglycemia incidences and the extent of an individual GV using different approaches. Recently, because of the ubiquitous nature of self-management mHealth apps, sensors and wearables have paved the way for the continuous accumulation of self-collected health data, which in turn contributed for the widespread research of machine learning applications in these tasks. In the reviewed articles, generally, the anomaly classification and detection approaches could be categorized as either model (process)–based or classifier (rule)–based approaches. Hypoglycemia classification and detection has been given more attention than hyperglycemia and GV detection, which might be because of its serious complication and the comparable complexity involved. The state-of-the-art indicates that various classes of machine learning have been developed and tested in these tasks. Regarding hypoglycemia classification and detection, feedforward ANNs, hybrid systems, SVM, DT, GA, adaptive neural fuzzy inference system, NARX, and NAR, Gaussian process regression, DBN, and BNN have been developed and tested. These techniques have explored various kinds of input parameters, notably BG, heart rate, QT interval, insulin, diet, physical activity, galvanic response, and skin impedance. Concerning hyperglycemia classification and detection, RNN, GP, HMM, feedforward ANN, GA, and hybrid systems have been developed and tested, exploring various types of input parameters including BG, insulin, heart rate, and QT interval. GV detection is a recent development, which has great importance in quantifying factors associated with hypoglycemia and hyperglycemia incidence. In this regard, there is some research and development involving machine learning techniques, for example, the feedforward ANN, NBC, and SVM.

Most of these studies have used a theoretical threshold suggested either by the literature or physicians and various concerned bodies such as the American Diabetes Association. However, the problem here is that some patients might feel no symptoms at the specified threshold, and it may vary from patient to patient. Therefore, a model should consider such differences among the patients (intra- and intervariability) and also track its temporal change over time. Moreover, the studies should give more emphasis on the TL and various types of inputs used. Furthermore, researchers should give proper emphasis to develop anomaly classification and detection models, which are capable of justifying and reporting the underlying cause, as either due to patient controllable or patient uncontrollable parameters. Generally, we foresee that these developments might encourage researchers to further develop and test these systems on a large-scale basis.

## Appendix

Multimedia Appendix 1 Analysis of reported parameters, data characteristics, class of machine learning, and performance metrics.

Multimedia Appendix 2 Detail on reported accuracy, inputs and performance metrics used, and machine learning categorization.

## Abbreviations

ANFIS: adaptive neural fuzzy inference system
ANN: artificial neural network
AR: autoregressive
BBNN: block-based neural network
BG: blood glucose
BNN: Bayesian Neural Network
CGM: continuous glucose monitoring.
DBN: deep belief network
DT: decision tree
EA: evolutionary algorithm
ECG: electrocardiogram
ELM: extreme learning machine
GA: genetic algorithm
GP: genetic programming
GV: glycemic variability
HMM: hidden Markov model
MLP: multilayer perceptron
NAR: nonlinear autoregressive network
NARX: nonlinear autoregressive network with exogenous inputs
NBC: Naive Bayes classifier
POC: point-of-care
RF: random forest
RNN: recurrent neural network
ROC: receiver operating characteristic
SLP: single-layer perceptron
SMBG: self-monitoring blood glucose
SVM: support vector machine
VTWNN: variable translation wavelet neural network
