# Supplementary material for: Data-Driven Blood Glucose Pattern Classification and Anomalies Detection: Machine-Learning Applications in Type 1 Diabetes
Source: J Med Internet Res. 2019 May 1;21(5):e11030. doi: 10.2196/11030 (PMC6658321; doi:10.2196/11030)
Supplement: Multimedia Appendix 1 [file jmir_v21i5e11030_app1.pdf]

Multimedia Appendix 1: Analysis of reported parameters, data characteristics, machine learning class, and performance metrics

Table 1: Reported input features, machine learning class and accuracy.

Study	Features								Type of Machine Learning																			Performance		
	BG	Insulin	Phy. Activity	Diet	HR	QT interval	Skin Impedance	Galvanic response	DT	SVM	RNN	ANN	NARX & NAR	Fuzzy	NBC	Rule Based	ELM	HMM	DBN	Gaussian Pro.	BBNN	VTWNN	BNN	CNLN	GP	GA	Hybrid	Sensitivity	Specificity	Accuracy
[25]	■				■	■						■														■	■	76%	58%	--
[26]& [27]	■				■	■						■				■										■	■	79%	52%	--
[28]	■								■																			86%	96%	96%
[29]	■	■		■								■	■															80%	98%	98%
[30]	■	■	■	■						■		■								■								94%	--	--
[31]	■	■		■						■										■								--	--	88%
[32]	■				■		■					■		■														--	--	--
[33] & [34]	■									■																		78%	96%	--
[35] & [36]	■									■																		81%	93%	--
[37]	■			■					■																			80%	80%	80%
[38]	■								■	■																		--	--	68%
[39]					■	■						■																60%	100%	85%
[40] & [41] & [42]	■				■	■								■												■		75%	50%	--
[43]	■				■	■						■		■													■	86%	80%	--
[44]	■				■	■					■																■	77%	53%	--
[45]	■				■	■							■			■								■				79%	54%	--
[46]	■				■	■											■											78%	60%	--
[47]	■	■		■	■		■	■		■																		--	--	--
[48]	■				■	■						■		■												■		80%	73%	--
[49]	■																■											--	95%	--
[50]	■				■	■	■					■																95%	41%	--
[51] & [52] & [53]	■				■	■	■																■					83%	64%	--
[54] & [55]	■				■							■																82%	63%	--
[56]	■				■							■														■	■	75%	60%	--
[57]	■				■	■				■																		82%	60%	--
[58] & [59]	■				■	■				■				■													■	74%	59%	63%
[60] & [61]	■				■	■				■				■													■	75%	83%	--
[62]	■				■	■					■																	77%	52%	--
[63] & [64]	■				■	■																■						79 %	50 %	--
[65] & [66]	■				■	■															■							77%	51%	--
[67]	■				■	■								■														79%	52%	--
[68]	■				■	■																		■				77%	55%	--

[69]																												84%	52%	--
[70]																												80%	50%	--
[71]																												78%	60%	--
[72]																												--	90%	86%
[73]																												75%	55%	--
[74]																												--	--	100%
[75]																												--	--	100%
[15]																												--	--	--
[76]																												71%	65%	--
[77]																												--	--	--
[78]																												--	--	85%
[79]																												97%	74%	90%
[80]																												87%	97%	94%
[81]																												72.23%	92%	90%
[82]																												73%	60%	--

Data Characteristics and Input Parameters

Input Parameters

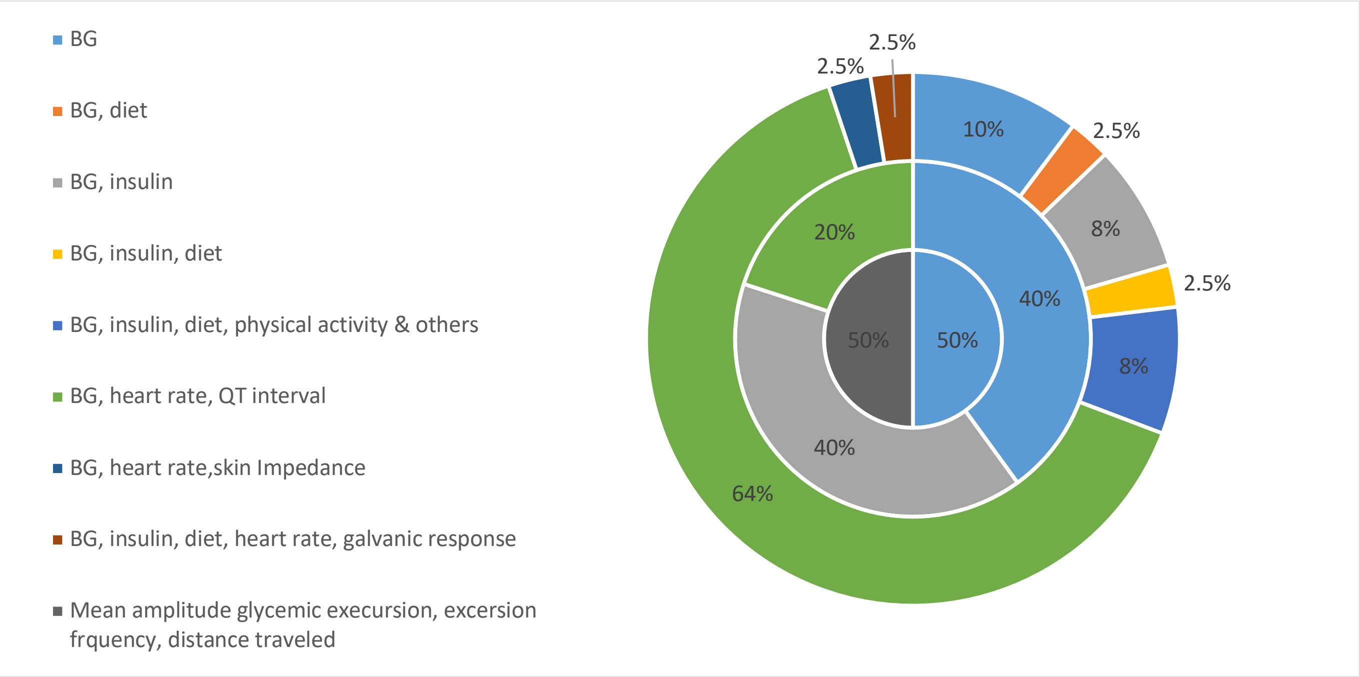

Figure 5: Types of input variables used in the studies. The outer ring, middle ring and inner rings depicts the type of input variables used in hypoglycaemia, hyperglycaemia and glycaemic variability classification and detection algorithms.

Class of machine learning

Hypoglycemia classification & detection

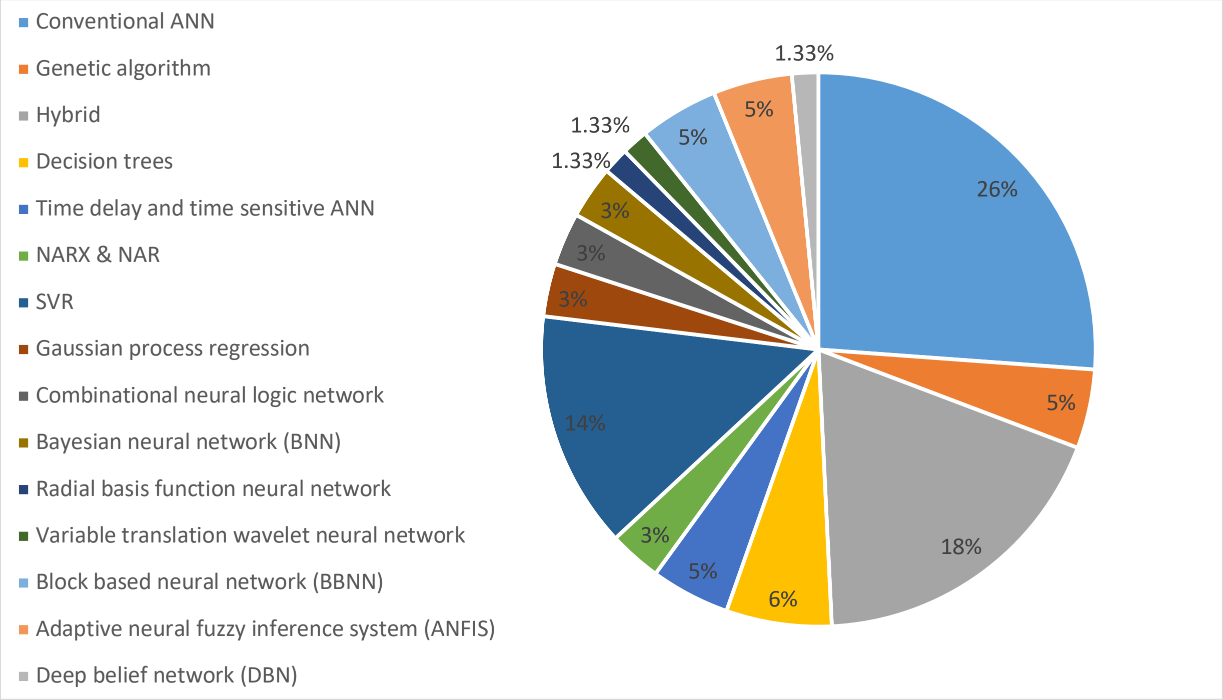

Figure 6: Classes of machine learning used in hypoglycaemia classification and detection.

Hyperglycemia classification & detection

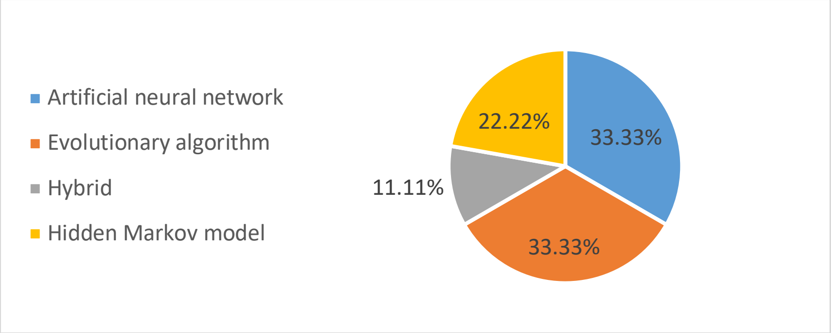

Figure 7: Classes of machine learning used in hyperglycaemias classification and detection.

Glycemic variability classification & detection

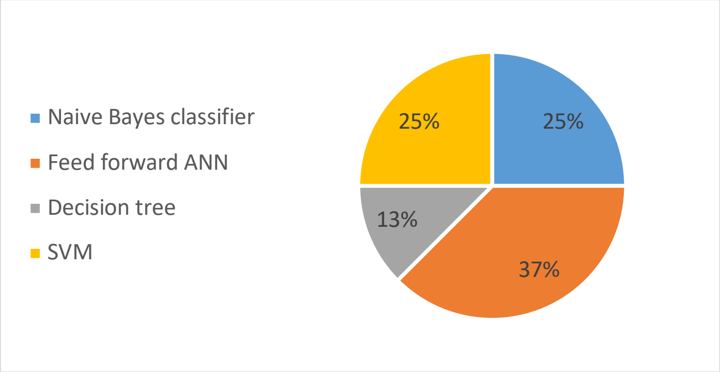

Figure 8: Classes of machine learning used in glycaemic variability classification and detection.

Performance metrics

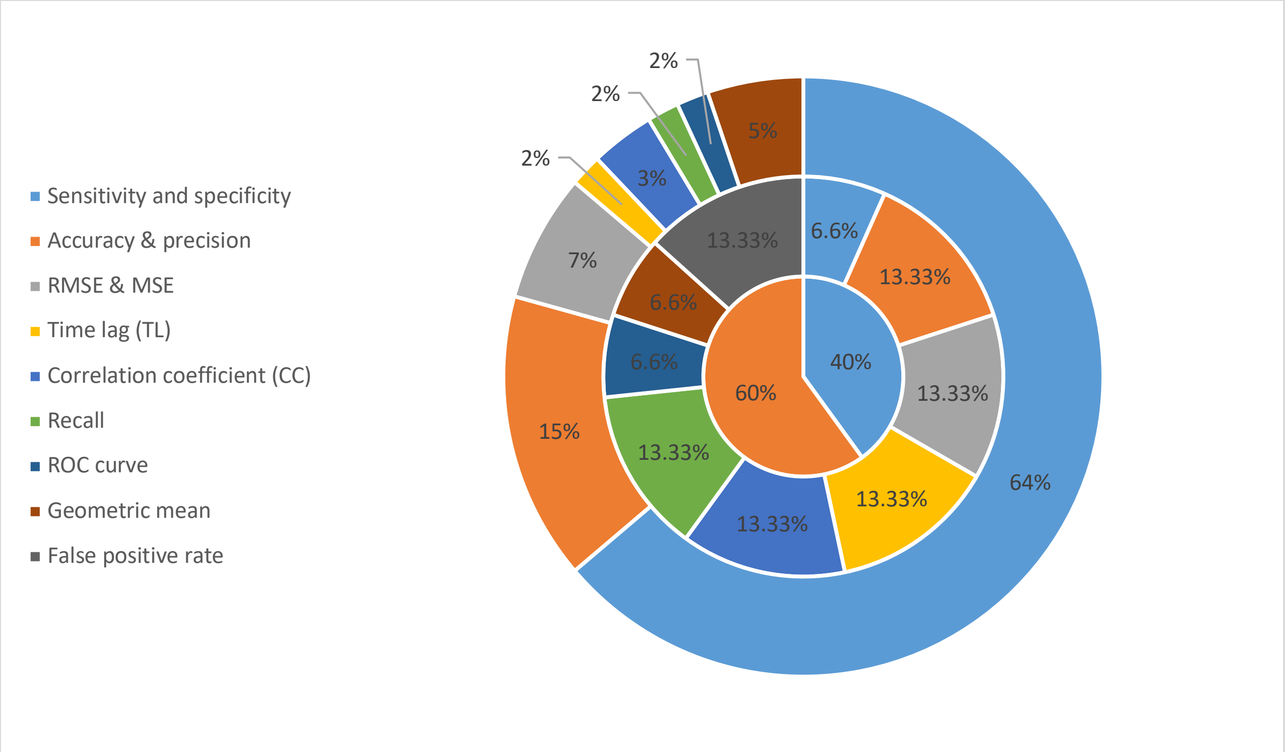

Figure 9: Depicts the type of performance metrics used in the studies. The outer ring, middle ring and inner rings depicts the type of performance metrics used in hypoglycaemia, hyperglycaemias and glycaemic variability classification and detection algorithms.
