# Supplementary material for: Data-Driven Blood Glucose Pattern Classification and Anomalies Detection: Machine-Learning Applications in Type 1 Diabetes
Source: J Med Internet Res. 2019 May 1;21(5):e11030. doi: 10.2196/11030 (PMC6658321; doi:10.2196/11030)
Supplement: Multimedia Appendix 2 [file jmir_v21i5e11030_app2.pdf]

Multimedia Appendix 2: Detail on reported accuracy, inputs and performance metrics used, and machine learning categorization.

Table 1: Data extracted from the literatures included in the study.

Ref.	Subject	Type of input	Data Format/Data source	Input Pre-processing	Class of Machine Learning	Performance Metrics
[1]	16 Real (14.6±1.5 years of age)	BG, rate of change of heart rate, Corrected QT interval of electrocardiogram signal, Rate of change of corrected QT interval	Department of Health, Government of Western Australia, Yellow Spring Instrument for BG	N/A	Hybrid-(feed-forward neural network and genetic algorithm)	Sensitivity and specificity
[2] & [3]	16 Real (14.6±1.5 years of age)	BG, rate of change of heart rate, Corrected QT interval of electrocardiogram signal, Rate of change of corrected QT interval	HypoMon (Hypoglycemia Monitor from AIMedics Pty, Ltd.) to measure the required physiological parameters, Yellow Spring Instrument to measure BG.	N/A	Hybrid-(feed-forward neural network, rule discovery, and genetic algorithm)	Sensitivity and specificity
[4]	10 Real (2 male & 8 female, 11-70 years of age)	BG (past and present), Rate of change of BG	American Hospital in Dubai-Medtronic CGM & Insulin Pump	Differencing BG Values	Decision trees	Accuracy, sensitivity and specificity
[5]	10 Real (2 male & 8 female, 11-70 years of age)	Subcutaneous glucose measurements, insulin, and carbohydrate intake	American Hospital in Dubai-Medtronic CGM & Insulin Pump	N/A	Artificial Neural Networks (ANN), Time- Sensitive ANN (TS-ANN), Time Delay Neural Network (TDNN), Nonlinear Autoregressive Network with exogenous inputs (NARX), Distributed Time Delay Neural Network (DTDNN), and Nonlinear Autoregressive Network (NAR).)	Root mean squared error, sensitivity, specificity, and Accuracy
[6]	15 Real (3 women and 12 men whose ages ranged from 19 to 65 years (average, 40.3 – 13.5 years))	Glucose profile, meals, insulin intake, and physical activities (exercise), sleep	Guardian Real-time CGM system (Medtronic Minimed Inc., Northridge, CA), SenseWear armband (BodyMedia Inc., Pittsburgh, PA) physical activity monitor	N/A	Support vector for regression (SVR), feed-forward multilayer perceptron (MLP) and Gaussian processes (GP) regression	Sensitivity, precision, and time lag
[7]	15 Real	Glucose, rate of meal glucose appearance, plasma insulin concentration, meal-derived glucose inserted in plasma, energy expenditure, hour of day	Guardian Real-time CGM system (Medtronic Minimed Inc., Northridge, CA), SenseWear armband (BodyMedia Inc., Pittsburgh, PA) physical activity monitor	N/A	Support vector regression (SVR) or Gaussian processes (GP) regression	Standard deviation of the RMSE and correlation coefficient, sensitivity
[8]	12 real (6 normal & 6 T1DM)-6 males & 6 females aged 26 ± 3 years)	Measured BGLs, skin impedances, heart rates	N/A	Normalization	A novel fuzzy neural network estimator algorithm (FNNE)-a parallel combination of fuzzy inference mechanism (FIM) and a multi-layered neural network	Mean square error, correlation coefficient
[9] & [10]	10 Real (all male, age 44 ± 15 years)	Current CGM reading, first derivative of the current CGM reading and the reading before, Time since last insulin injection, Linear regression, Skewness, Kurtosis of the CGM readings in multiple intervals,	CGM device (Guardian RT®, Minimed Inc., USA), emoCue Glucose 201+ glucose analyzer (HemoCue®, Ängleholm, Sweden)	Reconstruction of CGM data using spline interpolation and a rough feature elimination, using fast SEPCOR algorithm.	Support vector machine	Sensitivity, and specificity
[11] & [12]	10 Real (all male, age 44 ± 15 years)	Current CGM reading, first derivative of the current CGM reading and the reading before, Time since last insulin injection, Linear regression, Skewness, Kurtosis of the CGM readings in multiple intervals,	CGM device (Guardian RT®, Minimed Inc., USA), emoCue Glucose 201+ glucose analyzer (HemoCue®, Ängleholm, Sweden)	Reconstruction of CGM data using spline interpolation and a rough feature elimination, using fast SEPCOR algorithm.	Support vector machine	Sensitivity, and specificity
[13]	21 Real	BG, Meal, Rate of decrease from a peak and absolute level of the BG at the decision point	Diagnostic (professional) CGM devices	N/A	Decision trees	Accuracy, sensitivity, and specificity
[14]	1 Real (Male)	Glucose levels right before meals (G1), Glucose levels after more than 5 hours (G2), Time interval (T), Average Fasting glucose level (AG1), The rate of decrease in [Glu], Ratio of current level to average	Self-Monitored Blood Glucose (SMBG)	Extraction and representing temporal change information,	Decision tree and SVM	Accuracy
[15]	8 Real (five male, three female aged 35 +_ 13.5 years (mean +-SD))	ECG Signal information	Portable apparatus was developed to record the EEG-To record the EEG signal, a digitalization module was developed that was linked to a palmtop PC,	Amplitude Normalization and Transformation (FFT).	Artificial neural networks (MLP-ANNs)	Accuracy, sensitivity, and specificity
[16] & [17] & [18]	16 Real (children-14.6±1.5 years)	BG, heart rate (HR), corrected QT interval of the ECG signal, change of HR, and the change of corrected QT interval	skin surface biosensor electrodes for the measurement of physiological parameters, Yellow Spring Instruments (YSI) used for collection of reference BG	Normalization and linear correction analysis..	Genetic algorithm (GA)-based multiple regression with fuzzy inference system (FIS)	Sensitivity and specificity
[19]	16 Real (children-14.6±1.5 years)	BG, heart rate and the corrected QT interval of the electrocardiogram (ECG) signal	skin surface biosensor electrodes for the measurement of physiological parameters, Yellow Spring Instruments (YSI) used for collection of reference BG	Normalization	Hybrid particle-swarm-optimization-based fuzzy-reasoning model, Feed-Forward Neural Network (FFNN)	Sensitivity and specificity
[20]	15 Real (children-14.6±1.5 years)	BG, heart rate and the corrected QT interval of the electrocardiogram (ECG) signal	skin surface biosensor electrodes for the measurement of physiological parameters, Yellow Spring Instruments (YSI) used for collection of reference BG	Normalization and Partitioning of the input.	Hybrid rough set based neural network (RNN)	Sensitivity and specificity
[21]	15 Real (children-14.6±1.5 years)	BG, electrocardiogram signal (heart rate (HR) & corrected QT interval (QTc), change of heart rate (HR) and corrected QT interval (QTc))	skin surface biosensor electrodes for the measurement of physiological parameters, Yellow Spring Instruments (YSI) used for collection of reference BG	Correlation analysis	Multiple regression (MR)-based combinational neural logic approach - Combinational neural logic network (NLN-feedforward neural network (FFNN) & rule based logic)	Sensitivity and specificity
[22]	16 Real (children-14.6±1.5 years)	BG, heartrate (HR), corrected QT interval of ECG (QTc),change of heartrate (ΔHR), and change of corrected QT interval of ECG(ΔQTc),	skin surface biosensor electrodes for the measurement of physiological parameters, Yellow Spring Instruments (YSI) used for collection of reference BG	Normalization	Single-hidden Layer Feedforward neural Network (SLFN) with L hidden nodes (Extreme learning machine(ELM)-based neural network)	Sensitivity and specificity
[23]	1 Real (middle-aged male)	BG, Insulin, meals, heart rate (HR), galvanic skin response (GSR), and skin and air temperatures (ST and AT).	Medtronic insulin pump & Dexcom CGM system, smart phone (reported meals, sleep & exercise), Fitness band, Basis Peak (heart rate (HR), galvanic skin response (GSR), & skin and air temperatures (ST & AT))	Feature extraction and selection- using greedy feature selection	Support vector machines (SVM)	Sensitivity, specificity, precision and recall
[24]	5 real	BG, heart rate(HR),corrected QT interval, change of HR and change of corrected QT interval	N/A	Linear correction analysis	Multiple regression with fuzzy inference system(FIS), Neural network. Parameters optimized through genetic algorithm (GA), particle-swarm optimization respectively.	Sensitivity and specificity
[25]	N/A	Blood Glucose (CGM)	N/A	N/A	Single-hidden Layer Feedforward neural Network (SLFN) with L hidden nodes (extreme learning machines (ELM) and regularized ELM (RELM))	Root mean square error (RMSE), sensitivity, specificity, ROC
[26]	21 Real (children, 14.4±1.6 years)	BG, heart rate, corrected QT interval of the ECG signal and skin impedance	HypoMon, blood glucose (BG) levels were collected as reference using Yellow Spring Instruments	Normalization	Feedforward multi-layer neural network	Sensitivity and specificity
[27] & [28] & [29]	16 Real (children, 14.4±1.6 years)	BG, heart rate, corrected QT interval of the ECG signal and skin impedance	HypoMon, actual blood glucose (BG) levels were collected as reference using Yellow Spring Instruments	Normalization	Feed-forward multi-layer neural network (Bayesian neural network-Bayesian learning)	Sensitivity and specificity
[30] & [31]	5 real (adolescent patients between the ages of 12 and 18 year old)	BG, EEG responses-the centroid theta frequency and the centroid alpha frequency from each channel	Compumedics system, electromyogram (EMG) signals, electrooculogram (EOG) signals, BGLs were acquired using Yellow Spring Instruments	Filtering (IIR highpass filter and A notch filter at 50Hz)	Feed-forward multi-layer neural network (standard particle swarm optimization strategy is applied to optimize the parameters)	Sensitivity and specificity, ROC Curve
[32]	5 real (adolescent patients between the ages of 12 and 18 year old)	BG, EEG responses-the centroid theta frequency and the centroid alpha frequency from each channel	Compumedics system, electromyogram (EMG) signals, electrooculogram (EOG) signals, BGLs were acquired using Yellow Spring Instruments	Filtering (IIR highpass filter), Feature extraction (Fast Fourier Transform (FFT))	Feed-forward multi-layer neural network (genetic algorithm and Levenberg Marquardt algorithm)	Sensitivity and specificity, ROC Curve
[33]	5 real	BG, Repolarization variabilities- QTeVI, TpTeVI, ToTeVI and RTpVI	Compumedics system, BGLs were acquired using Yellow Spring Instruments	N/A	Swarm-based Support vector machine (SVM) (Radial basis function (RBF)-standard particle swarm optimization strategy is applied to optimize the parameters)	Sensitivity, specificity, and geometric mean
[34] & [35]	5 real (with age of 16±0.7 years)	BG, heart rate, corrected QT (QT c) interval and corrected TpTe (TpTec) interval	Compumedics system, BGLs were acquired using Yellow Spring Instruments	Feature extraction	Fuzzy Support Vector Machine - kernel functions (radial basis function (RBF), exponential radial basis function (ERBF) and polynomial function)	Sensitivity, specificity and accuracy
[36] & [37]	5 real	BG, HR, RTpc, QTc, TpTec, ToTec and QTpc	Princess Margaret Hospital in Perth, Australia, with approval from Women's and Children's Health Service,	Normalization and feature extraction	Hybrid particle swarm - based fuzzy support vector machine (SFisSvm) technique- kernel functions (radial basis function	Sensitivity, specificity and geometric mean

			Department of Health, Government of Western Australia		(RBF), exponential radial basis function (ERBF) and polynomial function)-hybrid particle swarm optimization	
[38]	15 Real (children)	BG, heart rate (HR), corrected QT (QTc), change in the heart rate (ΔHR) and change in the QTc interval (ΔQTc)	Compumedics system, BGLs were acquired using Yellow Spring Instruments	Normalization	Hybrid particle swarm optimization based normalized radial basis function neural network (NRBFNN)- hybrid particle swarm optimization with wavelet mutation (HPSOWM)	Sensitivity and specificity
[39] & [40]	15 Real (children)	BG, heart rate (HR) and corrected QT interval (QTc)	Compumedics system, BGLs were acquired using Yellow Spring Instruments	Normalization	Variable translation wavelet neural network (VTWNN)- hybrid particle swarm optimization with wavelet mutation (HPSOWM)	Sensitivity and specificity
[41] & [42]	15 Real (children)	BG, heart rate (HR) and the corrected QT interval (QTc)	Compumedics system, BGLs were acquired using Yellow Spring Instruments	Normalization	Evolvable block based neural network (BBNN)- hybrid particle swarm optimization with wavelet mutation (HPSOWM)	Sensitivity, specificity, ROC Curve, and geometric mean value
[43]	15 Real (children)	BG, heart rate (HR) and corrected QT (QTc)	Compumedics system, BGLs were acquired using Yellow Spring Instruments	N/A	Adaptive neural fuzzy inference system (ANFIS)- hybrid particle swarm optimization with wavelet mutation (HPSOWM)	Sensitivity and specificity
[44]	15 Real (children with ages 14.6 ± 1.5 years)	BG, heart rate (HR) and the corrected QT interval (QTc)	N/A	N/A	Combinational neural logic network (NLN) - hybrid particle swarm optimization with wavelet mutation (HPSOWM)	Sensitivity and specificity
[45]	15 Real (children with ages 14.6 ± 1.5 years	BG, HR, QTc, change in HR and change in QTc	Compumedics system, BGLs were acquired using Yellow Spring Instruments	Rough Set based Pre-processing	Hybrid rough-block-based neural network (R-BBNN)- hybrid particle swarm optimization with wavelet mutation (HPSOWM)	Sensitivity and specificity
[46]	15 Real (children with ages 14.6 ± 1.5 years	BG, heart rate (HR) and corrected QT (QTc)	Compumedics system, BGLs were acquired using Yellow Spring Instruments	Feature representation using unsupervised restricted Boltzmann machines (RBM)	Deep belief network (DBN) block based neural network (BBNN)- greedy layer-wise manner training-backpropagation of error derivatives.	Sensitivity and specificity
[47]	16 Real (children with ages 14.6 ± 1.5 years	BG, HR, QTc, Change in HR and Change in QTc	N/A	N/A	ELM trained feed-forward neural network (ELM-FFNN)- single hidden layer feedforward neural network (FFNN)- Extreme Learning Machine (ELM)	Sensitivity and specificity
[48]	N/A	BG levels, Slope of changes in BG levels, dose response to IV insulin, Insulin titration promptness, Cumulated, administered insulin	MIMIC II (Multiparameter Intelligent Monitoring in Intensive Care database II) from the ICUs at Beth Israel Deaconess Medical Center, Boston, MA.	Feature selection and ranking	Decision tress (Classification tree-C5.0)	Sensitivity and specificity
[49]	16 Real (children with ages 14.6 ± 1.5 years	BG, heartrate (HR), corrected QT interval of the electrocardiogram (ECG) signal (QTc), change of HR, and change of QTc	Princess Hospital for Children in Perth, Western Australia, Australia. The actual BG levels were collected as reference using the Yellow Spring Instruments	N/A	Fuzzy inference system (FIS)- multi-objective optimization approach- wavelet mutated differential evolution optimizers	Sensitivity and specificity
[50]	23 real (17 to 70 years of age)	Glucose and insulin	Medtronic insulin pumps (Medtronic MiniMed Inc., Northridge, CA, USA) combined with a real-time CGM system under normal daily living conditions	Smoothening and Filtering	Adaptive data-driven models (autoregressive with output correction – cARX, & a recurrent neural network – RNN)- Data fusion techniques (Dempster-Shafer Evidential Theory (DST),Genetic Algorithms (GA), & Genetic Programming (GP)- teacher-forced, real-time, recurrent learning algorithm	RMSE, time lag (TL), correlation coefficient, and receiver operating characteristic (ROC) curve
[51]	23 real (17 to 70 years of age)	Glucose and insulin	Medtronic insulin pumps (Medtronic MiniMed Inc., Northridge, CA, USA) combined with a real-time CGM system under normal daily living conditions	N/A	Hybrid-autoregressive with an output correction module/recurrent neural network (cARN)-based EWS, recurrent neural network (RNN)- teacher-forced, real-time, recurrent learning algorithm	Root mean square error, TL, and correlation coefficient
[52]	N/A	BG	N/A	N/A	Hidden Markov model (HMM)- Baum-Welch algorithm that belongs to the family of Expectation Maximization algorithms, Forward-Backward algorithm	Precision, false positives rates, Recall
[53]	10 real (adolescent)	BG, HR, PR, QTC, RTC, TPTEC, time domain (Mean RR interval (MeanRR), standard deviation of the RR interval index (SDNN), root mean square of successive RR interval differences(RMSSD), Percentage of consecutive RR intervals that differ by more than 50 ms (pNN50), HRV triangular index (HRVi), Baseline width of the RR interval histogram evaluated through triangular interpolation (TINN)) & frequency domain, Total spectral power (TotalPw), Ratio between LF and HF components (LF/HF))	During the study period, ECG signals were continuously recorded by a medical device called Compumedics with the sampling rate of 512 Hz, while actual blood glucose was collected as reference using Yellow Springs Instruments. Kubios HRV Analysis Software package	Normalization & interpolation	Multilayer feed-forward neural network- Levenberg-Marquardt (LM) algorithm-error back propagation learning method	Sensitivity & specificity, geometric mean.
[54]	N/A	BG	N/A	N/A	Hidden Markov model (HMM)- Baum-Welch algorithm that belongs to the family of Expectation Maximization algorithms, Forward-Backward algorithm	Precision, false positives rates, recall
[55]	11 patients (9 female and 2 male patients ranged in age from 26 to 67 years)	Mean amplitude of glycemic excursion (MAGE), i) excursion frequency. ,ii) distance traveled and along with physicians' variability classification for that day	Medtronic Paradigm® insulin pumps with Real-Time continuous glucose monitors. GlycoMark™. The CGM data were extracted from the Medtronic CareLink® database into clinical diabetes research database.	N/A	Naive Bayes classifier (probabilistic reasoning), a multilayer perceptron (ANN), and a logistic model tree (decision tree built using logistic regression)	Accuracy (classifier vs. physician glycemic variability classifications of daily CGM charts)
[56]	19 patients (14 female and 5 male patients, ranging in age from 17 to 71 (mean 47) years)	MAGE, Excursion Frequency (EF), Distance Traveled (DT), SD, Area Under the Curve, Central Image Moments, Eccentricity, Discrete Fourier Transform, Roundness Ratio, Bending Energy, Direction Codes, Maximum Slope	Medtronic Paradigm® insulin pumps with Real-Time continuous glucose monitors. GlycoMark™. The CGM data were extracted from the Medtronic CareLink® database into our clinical diabetes research database.	Smoothing and feature selection (greedy forward selection and greedy backward elimination)	Multilayer perceptrons (MPs) and support vector machines for regression (SVR)- Gaussian kernel, Back propagation	Accuracy, sensitivity, and specificity
[57]	11 patients (9 female and 2 male patients ranged in age from 26 to 67 years)	Minimum Input-Direction Codes, Excursion Frequency, Standard Deviation, and Distance Traveled	Medtronic Paradigm® insulin pumps with Real-Time continuous glucose monitors. GlycoMark™. The CGM data were extracted from the Medtronic CareLink® database into clinical diabetes research database.	Smoothing using cubic splines & feature selection (Pearson's Correlation Coefficient & t-test, wrapper approach using greedy backward elimination)	Naive Bayes (NB), Multilayer Perceptron (MP), and Support Vector Machine (SVM)- Gaussian kernel, Back propagation, grid search optimization	Accuracy, sensitivity, and specificity
[58]	6 real patients (Two male, and four female)	SMBG (finger sticks), CGM, Insulin, Physical activity, Diet, illness and other life events	OhioT1DM Dataset (Medtronic 530G insulin pumps, Medtronic Enlite CGM sensors, Reported life-event data via a custom smartphone app and provided physiological data from a Basis Peak fitness band.)	N/A	Artificial Neural Network (ANN)	Accuracy, sensitivity, and specificity
[59]	8 real patients (12 and 18 years age)	Frequency features of occipital lobe (Centroid frequency and spectral entropy)	Yellow Spring Instruments for BG and Compumedics System for EEG	Fast Fourier Transform	Bayesian regularized neural network	Sensitivity and specificity

Table 2: Reported accuracy from the literatures.

Ref.	Reported System performance (Accuracy, Sensitivity, Specificity & Time horizon)	Comment
[1]	Neural network trained with Genetic algorithm (NN-GA) (Sensitivity (75.57%), Specificity (57.68%))	Compared with Neural network trained with a Levenberg Marquardt (LM) algorithm, Statistical Regression (SR), Fuzzy Regression (FR), Genetic Programming (GP), and Genetic Programming based Fuzzy Regression (GP-FR) for detecting hypoglycemia incidences.
[2] & [3]	GA-NN based Rule discovery (Specificity (79.11%) and Sensitivity (52.01%))	Compared with Fuzzy regression (FR), Genetic programming (GP), fuzzy regression based Genetic programming (FR-GP), neural network trained with back propagation (NN-BP) and Neural network trained genetic algorithm (NN-GA) for hypoglycemia detection.
[4]	Decision tree - 30 min Hypoglycemia prediction-(Sensitivity (86.47%), Specificity (96.22), Accuracy (95.97%))	Compared various decision tree approach J4.8, REPTree, Bagging, J4.8 and cost sensitive version of J4.8 using CGM data.
[5]	Time-Sensitive ANN (TS-ANN) - 30 min hypoglycemia prediction-(average specificity (98.2%), average accuracy (97.6%) and average sensitivity (80.19%) with a maximum value reaching 93%).	Hypoglycemia were detected by BG prediction using neural network NARX (Nonlinear Autoregressive network with Exogenous Inputs) trained with Bayesian Regularization back propagation training Algorithm.
[6]	Support vector for regression - Free-living conditions (Nocturnal hypoglycemic – Sensitivity (30-min - 94% & 60-min - 94%) with time lags of 5.43 min and 4.57 min, respectively. Diurnal – without physical activities sensitivity (30-min - 92% and 60-min	Compared Support vector for regression, Multilayer perceptron, and Gaussian processes for prediction of nocturnal and diurnal hypoglycemic events. The study also investigated the effect of Physical activity information.

	-96%), with both time lags being less than 5 min. Diurnal – with physical activities decreases the sensitivity by 8% and 3%, respectively. Both nocturnal and diurnal predictions show a high (> 90%) precision.	
[7]	SVR - (30min- Average prediction Accuracy Hypo (87%) & Hyper (96%), 60min- Hypo (83%) & Hyper (94%)). GP (30min- Average prediction Accuracy Hypo (88%) & Hyper (95%), 60min- Hypo (85%) & Hyper (88%))	Compared support vector regression (SVR) and Gaussian process (GP).
[8]	Fuzzy neural network estimator algorithm (FNNE) predicted the onset of hypoglycemia episodes with a mean error of 0.071 (p < 0.03)	The FNNE algorithm was developed as a parallel combination of fuzzy inference mechanism (FIM) and a multi-layered neural network architecture.
[9] & [10]	Support vector regression (SVR) - with an event-based sensitivity of 100%, the algorithm produced only one false hypoglycemia detection. The sample-based sensitivity and specificity levels were 78% and 96%, respectively	Developed an android based system to detect hypoglycemia incidence using CGM and other information.
[11] & [12]	SVR with CGM- sample based Sensitivity 81%, and Specificity 93%	Compared CGM with and without SVR algorithm for hypoglycemia detection.
[13]	Classification and Regression Tree (CART) - Average accuracy (79.8%), average sensitivity (80.05%), overall specificity (79.9%)- The model was able to detect almost 80% of hypoglycemic events 15 min in advance	Investigated Classification and Regression Tree (CART) for hypoglycemia detection.
[14]	Decision tree (Accuracy (65.2%)) & Linear SVM (Accuracy (68.4%))	Investigated DT and SVM for hypoglycemia prediction.
[15]	ANN real time - Accuracy (85.2%), sensitivity (60%) and specificity (100%)	Invested real time and offline hypoglycemia detection using ECG signal.
[16] & [17] & [18]	Genetic algorithm based multiple regression with fuzzy inference - Sensitivity (75%) and specificity (over 50%)	Genetic algorithm is used to optimize regression and fuzzy rules. Compared various order multiple Regression Fuzzy Inference System and Linear multiple regression with various number of inputs.
[19]	Hybrid particle-swarm-optimization-based fuzzy-reasoning - Advanced hypoglycemic episodes (sensitivity (85.71%) & specificity (79.84%)) and hypoglycemic episodes (sensitivity (80.00%) & specificity (55.14%))	Investigated the applicability of PSO to optimize fuzzy rules and membership function of FRM. Compared with neural network and a regression method.
[20]	Hybrid rough set based neural network (RNN) - sensitivity (76.74%) and specificity (52.73%) whereas conventional FWNN with no rough approximation gives sensitivity (69.77%) and specificity (49.09%)	Hybrid particle swarm optimization with wavelet mutation (HPSOWM) is used to optimize RNN. Compared the result with a feedforward neural network (FWNN).
[21]	Combinational neural logic network with multiple regression - Sensitivity (79.07%) and Specificity (53.64%)	Hybrid particle swarm optimization with wavelet mutation (HPSOWM) is used to optimize the model parameters. Compared the result with neural logic network (NLN), wavelet neural network (WNN), feedforward neural network (FFNN), and multiple regression (MR)
[22]	Extreme learning machine (ELM)-based neural network -Sensitivity (78.00%) and Specificity (60.00%)	Compared the result with Particle swarm optimization based neural network (PSO-NN), Second order multiple regression fuzzy inference system (MR- FIS), Fuzzy inference system (FIS) and Linear multiple regression (LMR).
[24]	Fuzzy inference system with multiple regression - Sensitivity (80%) and Specificity (72.5%)	The fuzzy membership functions and rules are optimized using genetic algorithm. Compared the result with particle swarm optimization neural network.
[25]	Extreme learning machines (ELM)-the mean Specificity (95.4%) and the standard deviation (1.13)	Proposed and compared extreme learning machines (ELM) and regularized ELM (RELM) to predict hypoglycemia incidences using CGM readings.
[26]	Feedforward multi-layer neural network - Sensitivity (95.16%) and specificity (41.42%)	Proposed a neural network based hypoglycemia detection algorithm using ECG signal and skin impedance.
[27] & [28] & [29]	Bayesian neural network - Sensitivity (83.46%) and specificity (63.88%)	Investigated the applicability of Bayesian neural network to detect hypoglycemia from real time physiological parameters.
[30] & [31]	Particle Swarm Optimization-based Neural Network - Sensitivity (82%) and Specificity (63%)	Neural network parameters are optimized through PSO.
[32]	Neural network - Sensitivity (75%) and specificity (60%)	Investigated the possibility of combining Genetic Algorithm and Levenberg-Marquardt for neural network training in hypoglycemia detection algorithm.
[33]	Swarm-based support vector machine (SVM) - Sensitivity (82.14%) and Specificity (60.19%)	Investigated SVM-RBF for hypoglycemia detections and optimized the parameters through PSO.
[34] & [35]	Fuzzy Support Vector Machine (FSVM-RBF) - (Sensitivity (74.19%), Specificity (58.54%), Accuracy (63.20%))	Compared FSVM and SVM along with three different kernel functions (radial basis function (RBF), exponential radial basis function (ERBF) and polynomial function) for the classification purpose.
[36] & [37]	Hybrid particle swarm - based fuzzy support vector machine (SFisSvm) - Sensitivity (75.19%), Specificity (83.71%) and Geometric mean (79.33%)	The FIS and SVM parameters are optimized using a hybrid particle swarm optimization with wavelet mutation algorithm. The swarm based SVM uses RBF kernel (SSvmR), sigmoid kernel (SSvmS) and linear kernel function (SSvmL).
[38]	Normalized radial basis function neural network (NRBFNN) - Sensitivity (76.74%) and Specificity (51.82%)	The parameters of NRBFNN are optimized through hybrid particle swarm optimization with wavelet mutation (HPSOWM). Compared the result with radial basis function network, feedforward neural network, and multi regression.
[39] & [40]	Optimized variable translation wavelet neural network (VTWNN) - Sensitivity (79.07 %) and Specificity (50.00 %)	The parameters of VTWNN are optimized using a hybrid particle swarm optimization with wavelet mutation. Compared the result with wavelet neural network (WNN), feedforward neural network(FWNN2) and multi regression(MR)
[41] & [42]	Block Based Neural Network (BBNN) - Sensitivity (76.74%) and specificity (50.91%)	The BBNN parameters are optimized through a hybrid particle swarm optimization with wavelet mutation. Compared the result with feedforward neural networks and multiple regression.
[43]	Adaptive neural fuzzy inference system (ANFIS) – Sensitivity (79.09%) and specificity (51.82%)	The membership function and network parameters are optimized using swarm optimization with wavelet mutation (HPSOWM). Compared the result with fuzzy inference system (FIS), wavelet neural network (WNN), feedforward neural network (FWNN) and multiple regression (MR)
[44]	Combinational neural logic network (NLN) - Sensitivity (76.74%) and specificity (54.55%)	The NLN parameters are trained by hybrid particle swarm optimization with wavelet mutation (HPSOWM). Compared the result with neural logic network (NLN), wavelet neural network (WNN), feedforward neural network (FFNN) and multi regression (MR).
[45]	Hybrid rough-block-based neural network (R-BBNN) - Sensitivity (83.72%) and specificity (51.91%)	The R-BBNN parameters are optimized through a hybrid particle swarm optimization with wavelet mutation. Compared the result with BBNN, rough feedforward neural network (R-FWNN), wavelet neural network (WNN), SVM with a radial basis function and conventional feedforward neural network (FWNN).
[46]	Deep belief network (DBN) – Sensitivity (80.00%) and specificity (50.00%)	Compared the result with Block based neural network (BBNN), wavelet neural network (WNN), feedforward neural network (FFNN), and multiple regression (MR) models.
[47]	Extreme learning machine based feed-forward neural network (ELM-FFNN) - Sensitivity (78%) and specificity (60%)	Compared the result with multiple regression fuzzy inference system (MRFIS), Feed-forward neural network trained with particle swarm optimization (FFNN-PSO), Fuzzy inference system, and Linear multiple regression.

[48]	Classification tree - Predicted 82.12% of acute hypoglycemic events (specificity: 89.87%; positive predictive value: 88.72%; accuracy: 86.00%) and 76.99% of severe acute hypoglycemic events (80.53%, 74.31%, and 78.76% respectively).	Investigated towards predicting hypoglycemia incidence during intravenous (IV) insulin infusion for ICU patients.
[49]	Fuzzy inference system (FIS) - Sensitivity (75%) and Specificity (55%)	FIS parameters are tuned by an intelligent optimizer with two wavelet-mutated differential evolutions (WM-DE) engines. Compared the result with Neural network based rule discovery, linear multiple regression, evolved multiple regressions, feed-forward neural network (FFNN), and evolved fuzzy inference system.
[50]	Hypoglycemia (EWS (correct alarms=100%, detection time=16.7min, daily false alarms=0.08), EWS-DST (CA=100,DT=18.4min, DFA=1.0), EWS-GA(CA=100,DT=13min, DFA=0.17), EWS-GP(CA=100,DT=12.3min,DFA=0.17)), Hyperglycemia (EWS (CA=100,DT=14.7,DFA=0.8), EWS-DST (CA=100,DT=11.6min,DFA=0.73), EWS-GA (CA=100,DT=12.1min,DFA=0.73), EWS-GP(CA=100,DT=12min, DFA=0.33))	Investigated into advanced data fusion schemes for merging output of different hypo/hyperglycemia predictors such as Dempster-Schafer Evidential Theory and Evolutionary Methods (Genetic Algorithms, Genetic Programming). Compared the results with cARX and RNN models, and a linear fusion of the two.
[51]	ARX-based system - hypoglycemic (hyperglycemic) event prediction (accuracy of 100.0% (100.0%), detection time of 10.0 (8.0) min, and daily false alarms of 0.7 (0.5)). cARX-based system - Accuracy 100.0% (100.0%), DT 17.5 (14.8) min, & DFA 1.5 (1.3) and, RNN-based system Accuracy 100.0% (92.0%), DT 8.4 (7.0) min, and DFA 0.1 (0.2). The hybrid cARN-based EWS - 100.0% (100.0%) prediction accuracy, detection 16.7 (14.7) min in advance, and 0.8 (0.8) daily false alarms.	Investigated the performance improvement using a hybrid autoregressive with an output correction module/recurrent neural network (cARN). Compared performance of ARX, cARX, and RNN models.
[53]	Feed forward multi-layer neural network - Sensitivity (70.59%), specificity (65.38%) and geometric mean (67.94%)	Compared the ANN model with Linear Discriminant Analysis (LDA) and K-Nearest Neighbors (KNN) on hyperglycemia detection.
[52, 54]	Hidden Markov model (HMM) - The simulation result show that the proposed model is capable of detecting anomalies (i.e., no false positives) from the CGM readings based on historical data (in the presence of reasonable changes in the patient's daily routine).	Investigated the applicability of Hidden Markov model (HMM) in anomalies detection from the change in the patient's daily lifestyle.
[55]	Naïve Bayes classifier - matched the physicians' classifications 85% of the time that they were internally consistent and in agreement with each other.	Investigated into the applicability of characterizing blood glucose variability using new metrics with CGM data using Naïve Bayes classifier.
[56]	SVR models - When applied to 262 different CGM plots as a screen for excessive GV (accuracy (90.1%), sensitivity (97.0%), and specificity (74.1%).	Investigated the applicability of developing a perceived glycemic variability metric using SVM model. Compared the result with mean amplitude of glycemic excursion, standard deviation, distance travelled, and excursion frequency.
[57]	Multilayer Perceptron (MP)- (Accuracy 93.8%, Sensitivity 86.6%, Specificity 96.6%), Support Vector Machine (SVM)- (Accuracy 91.4%, Sensitivity 80.0%, Specificity 96.0%), Naive Bayes (NB) - (Accuracy 91.9%, Sensitivity 88.3%, Specificity 93.3%).	Investigated on an automatic glycemic variability detection and compared Naive Bayes (NB), Multilayer Perceptron (MP), and Support Vector Machine (SVM) models using CGM data.
[58]	Artificial Neural network – Average Accuracy (90%), Average sensitivity (72.23%) and Average specificity (92%)	Developed Artificial Neural Network integrated with physiological model for both blood glucose prediction and classification of hypoglycemia and further compared the result with existing models.
[59]	Bayesian regularized neural network - Sensitivity (73%) and specificity (60%)	Investigated and tested a feed-forward neural network trained with Bayesian regularization algorithm.

References

1. Chan, K.Y., S.H. Ling, T.S. Dillon, and H. Nguyen. *Classification of hypoglycemic episodes for Type 1 diabetes mellitus based on neural networks*. in *IEEE Congress on Evolutionary Computation*. 2010. Barcelona, Spain.
2. Chan, K.Y., S.H. Ling, T.S. Dillon, and H.T. Nguyen, *Diagnosis of hypoglycemic episodes using a neural network based rule discovery system.* Expert Systems with Applications, 2011. **38**(8): p. 9799-9808.
3. Kit Yan, C., L. Sai Ho, H.T. Nguyen, and F. Jiang. *A hypoglycemic episode diagnosis system based on neural networks for Type 1 diabetes mellitus*. in *2012 IEEE Congress on Evolutionary Computation*. 2012. Brisbane, QLD, Australia.
4. Eljil, K.S., G. Qadah, and M. Pasquier. *Predicting hypoglycemia in diabetic patients using data mining techniques*. in *2013 9th International Conference on Innovations in Information Technology (IIT)*. 2013. Abu Dhabi, United Arab Emirates.
5. Eljil, K.S., G. Qadah, and M. Pasquier, *Predicting Hypoglycemia in Diabetic Patients Using Time-Sensitive Artificial Neural Networks.* International Journal of Healthcare Information Systems and Informatics, 2016. **11**(4): p. 70-88.
6. Georga, E.I., V.C. Protopappas, D. Ardigo, D. Polyzos, and D.I. Fotiadis, *A glucose model based on support vector regression for the prediction of hypoglycemic events under free-living conditions.* Diabetes Technol Ther, 2013. **15**(8): p. 634-43.
7. Georga, E.I., V.C. Protopappas, S.G. Mougiakakou, and D.I. Fotiadis. *Short-term vs. long-term analysis of diabetes data: Application of machine learning and data mining techniques*. in *13th IEEE International Conference on BioInformatics and BioEngineering*. 2013. Chania, Greece.

8. Ghevondian, N., H.T. Nguyen, and S. Colagiuri. *A novel fuzzy neural network estimator for predicting hypoglycaemia in insulin-induced subjects*. in *2001 Conference Proceedings of the 23rd Annual International Conference of the IEEE Engineering in Medicine and Biology Society*. 2001. Istanbul, Turkey, Turkey.
9. Jensen, M.H., T.F. Christensen, L. Tarnow, Z. Mahmoudi, M.D. Johansen, and O.K. Hejlesen, *Professional Continuous Glucose Monitoring in Subjects with Type 1 Diabetes: Retrospective Hypoglycemia Detection.* Journal of Diabetes Science and Technology, 2013. **7**(1): p. 135-143.
10. Jensen, M.H., T.F. Christensen, L. Tarnow, M.D. Johansen, and O.K. Hejlesen, *An information and communication technology system to detect hypoglycemia in people with type 1 diabetes.* Studies in health technology and informatics, 2012. **192**: p. 38-41.
11. Jensen, M.H., T.F. Christensen, L. Tarnow, E. Seto, M. Dencker Johansen, and O.K. Hejlesen, *Real-time hypoglycemia detection from continuous glucose monitoring data of subjects with type 1 diabetes.* Diabetes Technol Ther, 2013. **15**(7): p. 538-43.
12. Jensen, M.H., Z. Mahmoudi, T.F. Christensen, L. Tarnow, E. Seto, M.D. Johansen, and O.K. Hejlesen, *Evaluation of an Algorithm for Retrospective Hypoglycemia Detection Using Professional Continuous Glucose Monitoring Data.* J Diabetes Sci Technol, 2014. **8**(1): p. 117-122.
13. Jung, M., Y.-B. Lee, S.-M. Jin, and S.-M. Park, *Prediction of Daytime Hypoglycemic Events Using Continuous Glucose Monitoring Data and Classification Technique.* arXiv preprint arXiv:1704.08769, 2017.
14. Jung, M. *Toward Designing Mobile Software to Predict Hypoglycemia for Patients with Diabetes*. in *2016 IEEE/ACM International Conference on Mobile Software Engineering and Systems (MOBILESoft)*. 2016. Austin, TX, USA.
15. Laione, F. and J. Marques, *Methodology for hypoglycaemia detection based on the processing, analysis and classification of the electroencephalogram.* Medical and Biological Engineering and Computing, 2005. **43**(4): p. 501-507.
16. Ling, S.H., H. Nguyen, and K.Y. Chan. *Genetic algorithm based fuzzy multiple regression for the nocturnal Hypoglycaemia detection*. in *IEEE Congress on Evolutionary Computation*. 2010. Barcelona, Spain.
17. Ling, S.S.H. and H.T. Nguyen, *Genetic-Algorithm-Based Multiple Regression With Fuzzy Inference System for Detection of Nocturnal Hypoglycemic Episodes.* IEEE Transactions on Information Technology in Biomedicine, 2011. **15**(2): p. 308-315.
18. Ling, S.H., H.T. Nguyen, and F.H.F. Leung. *Hypoglycemia detection using fuzzy inference system with genetic algorithm*. in *2011 IEEE International Conference on Fuzzy Systems (FUZZ-IEEE 2011)*. 2011. Taipei, Taiwan.
19. Ling, S.H. and H.T. Nguyen, *Natural occurrence of nocturnal hypoglycemia detection using hybrid particle swarm optimized fuzzy reasoning model.* Artif Intell Med, 2012. **55**(3): p. 177-84.
20. Ling, S.H., P.P. San, H.K. Lam, and H.T. Nguyen. *Non-invasive detection of hypoglycemic episodes in Type 1 diabetes using intelligent hybrid rough neural system*. in *2014 IEEE Congress on Evolutionary Computation (CEC)*. 2014. Beijing, China.
21. Ling, S.H., P.P. San, H.K. Lam, and H.T. Nguyen, *Hypoglycemia detection: multiple regression-based combinational neural logic approach.* Soft Computing, 2015. **21**(2): p. 543-553.
22. Ling, S.H., P.P. San, and H.T. Nguyen, *Non-invasive hypoglycemia monitoring system using extreme learning machine for Type 1 diabetes.* ISA Trans, 2016. **64**: p. 440-446.
23. Marling, C., L. Xia, R. Bunescu, and F. Schwartz. *Machine learning experiments with noninvasive sensors for hypoglycemia detection*. in *Proceedings of IJCAI 2016 Workshop on Knowledge Discovery in Healthcare Data*. 2016. New York, NY.
24. Mathews, S., *Fuzzy Inference System And Multiple Regression For Detection Of Hypoglycemia.* International Journal on Computational Science & Applications, 2012. **2**(2): p. 37-50.

25. Mo, X., Y. Wang, and X. Wu. *Hypoglycemia prediction using extreme learning machine (ELM) and regularized ELM*. in *2013 25th Chinese Control and Decision Conference (CCDC)*. 2013. Guiyang, China.
26. Nguyen, H.T., N. Ghevondian, and T.W. Jones. *Neural-Network Detection of Hypoglycemic Episodes in Children with Type 1 Diabetes using Physiological Parameters*. in *2006 International Conference of the IEEE Engineering in Medicine and Biology Society*. 2006. New York, NY, USA.
27. Nguyen, H.T., N. Ghevondian, and T.W. Jones. *Detection of nocturnal hypoglycemic episodes (natural occurrence) in children with Type 1 diabetes using an optimal Bayesian neural network algorithm*. in *2008 30th Annual International Conference of the IEEE Engineering in Medicine and Biology Society*. 2008. Vancouver, BC, Canada.
28. Nguyen, H.T., N. Ghevondian, S.T. Nguyen, and T.W. Jones. *Detection of Hypoglycemic Episodes in Children with Type 1 Diabetes using an Optimal Bayesian Neural Network Algorithm*. in *2007 29th Annual International Conference of the IEEE Engineering in Medicine and Biology Society*. 2007. Lyon, France.
29. Nguyen, H.T. and T.W. Jones. *Detection of nocturnal hypoglycemic episodes using EEG signals*. in *2010 Annual International Conference of the IEEE Engineering in Medicine and Biology*. 2010. Buenos Aires, Argentina.
30. Nguyen, L.B., A.V. Nguyen, S.H. Ling, and H.T. Nguyen. *A particle swarm optimization-based neural network for detecting nocturnal hypoglycemia using electroencephalography signals*. in *The 2012 International Joint Conference on Neural Networks (IJCNN)*. 2012. Brisbane, QLD, Australia.
31. Nguyen, L.B., A.V. Nguyen, S.H. Ling, and H.T. Nguyen. *An adaptive strategy of classification for detecting hypoglycemia using only two EEG channels*. in *2012 Annual International Conference of the IEEE Engineering in Medicine and Biology Society*. 2012. San Diego, CA, USA
32. Nguyen, L.B., A.V. Nguyen, S.H. Ling, and H.T. Nguyen. *Combining genetic algorithm and Levenberg-Marquardt algorithm in training neural network for hypoglycemia detection using EEG signals*. in *2013 35th Annual International Conference of the IEEE Engineering in Medicine and Biology Society (EMBC)*. 2013. Osaka, Japan.
33. Nuryani, S. Ling, and H.T. Nguyen. *Ventricular repolarization variability for hypoglycemia detection*. in *2011 Annual International Conference of the IEEE Engineering in Medicine and Biology Society*. 2011. Boston, MA, USA.
34. Nuryani, S.H. Ling, and H.T. Nguyen. *Hypoglycaemia detection for type 1 diabetic patients based on ECG parameters using Fuzzy Support Vector Machine*. in *The 2010 International Joint Conference on Neural Networks (IJCNN)*. 2010. Barcelona, Spain.
35. Nuryani, S.L. and H.T. Nguyen. *Electrocardiographic T-wave peak-to-end interval for hypoglycaemia detection*. in *2010 Annual International Conference of the IEEE Engineering in Medicine and Biology*. 2010. Buenos Aires, Argentina.
36. Nuryani, N., S.H. Ling, and H.T. Nguyen. *Hybrid particle swarm - based fuzzy support vector machine for hypoglycemia detection*. in *2012 IEEE International Conference on Fuzzy Systems*. 2012. Brisbane, QLD, Australia.
37. Nuryani, N., S.S. Ling, and H.T. Nguyen, *Electrocardiographic signals and swarm-based support vector machine for hypoglycemia detection.* Ann Biomed Eng, 2012. **40**(4): p. 934-45.
38. Phyo Phyo, S., S.H. Ling, and H.T. Nguyen. *Hybrid particle swarm optimization based normalized radial basis function neural network for hypoglycemia detection*. in *The 2012 International Joint Conference on Neural Networks (IJCNN)*. 2012. Brisbane, QLD, Australia.
39. Phyo Phyo, S., S.H. Ling, and H.T. Nguyen. *Optimized variable translation wavelet neural network and its application in hypoglycemia detection system*. in *2012 7th IEEE Conference on Industrial Electronics and Applications (ICIEA)*. 2012. Singapore, Singapore.

40. San, P.P., S.H. Ling, and H.T. Nguyen, *Hybrid PSO-based variable translation wavelet neural network and its application to hypoglycemia detection system.* Neural Computing and Applications, 2012. **23**(7-8): p. 2177-2184.
41. San, P.P., S.H. Ling, and H.T. Nguyen. *Block based neural network for hypoglycemia detection*. in *2011 Annual International Conference of the IEEE Engineering in Medicine and Biology Society*. 2011. Boston, MA, USA.
42. San, P.P., S.H. Ling, and H.T. Nguyen, *Industrial Application of Evolvable Block-Based Neural Network to Hypoglycemia Monitoring System.* IEEE Transactions on Industrial Electronics, 2013. **60**(12): p. 5892-5901.
43. San, P.P., S.H. Ling, and H.T. Nguyen. *Intelligent detection of hypoglycemic episodes in children with type 1 diabetes using adaptive neural-fuzzy inference system*. in *2012 Annual International Conference of the IEEE Engineering in Medicine and Biology Society*. 2012. San Diego, CA, USA.
44. San, P.P., S.H. Ling, and H.T. Nguyen. *Combinational neural logic system and its industrial application on hypoglycemia monitoring system*. in *2013 IEEE 8th Conference on Industrial Electronics and Applications (ICIEA)*. 2013. Melbourne, VIC, Australia
45. San, P.P., S.H. Ling, Nuryani, and H. Nguyen, *Evolvable Rough-Block-Based Neural Network and its Biomedical Application to Hypoglycemia Detection System.* IEEE Transactions on Cybernetics, 2014. **44**(8): p. 1338-1349.
46. San, P.P., S.H. Ling, and H.T. Nguyen. *Deep learning framework for detection of hypoglycemic episodes in children with type 1 diabetes*. in *2016 38th Annual International Conference of the IEEE Engineering in Medicine and Biology Society (EMBC)*. 2016. Orlando, FL, USA.
47. San, P.P., S.H. Ling, N.N. Soe, and H.T. Nguyen. *A novel extreme learning machine for hypoglycemia detection*. in *2014 36th Annual International Conference of the IEEE Engineering in Medicine and Biology Society*. 2014. Chicago, IL, USA
48. Zhang, Y. *Predicting occurrences of acute hypoglycemia during insulin therapy in the intensive care unit*. in *2008 30th Annual International Conference of the IEEE Engineering in Medicine and Biology Society*. 2008. Vancouver, BC, Canada.
49. Lai, J.C.Y., F.H.F. Leung, and S.H. Ling, *Hypoglycaemia detection using fuzzy inference system with intelligent optimiser.* Applied Soft Computing, 2014. **20**: p. 54-65.
50. Botwey, R.H., E. Daskalaki, P. Diem, and S.G. Mougiakakou. *Multi-model data fusion to improve an early warning system for hypo-/hyperglycemic events*. in *2014 36th Annual International Conference of the IEEE Engineering in Medicine and Biology Society*. 2014. Chicago, IL, USA
51. Daskalaki, E., K. Nørgaard, T. Züger, A. Prountzou, P. Diem, and S. Mougiakakou, *An Early Warning System for Hypoglycemic/Hyperglycemic Events Based on Fusion of Adaptive Prediction Models.* Journal of Diabetes Science and Technology, 2013. **7**(3): p. 689-698.
52. Zhu, Y., *Automatic detection of anomalies in blood glucose using a machine learning approach.* Journal of Communications and Networks, 2011. **13**(2): p. 125-131.
53. Nguyen, L.L., S. Su, and H.T. Nguyen. *Neural network approach for non-invasive detection of hyperglycemia using electrocardiographic signals*. in *2014 36th Annual International Conference of the IEEE Engineering in Medicine and Biology Society*. 2014. Chicago, IL, USA.
54. Zhu, Y. *Automatic detection of anomalies in blood glucose using a machine learning approach*. in *2010 IEEE International Conference on Information Reuse & Integration*. 2010. Las Vegas, NV, USA
55. Marling, C.R., J.H. Shubrook, S.J. Vernier, M.T. Wiley, and F.L. Schwartz, *Characterizing Blood Glucose Variability Using New Metrics with Continuous Glucose Monitoring Data.* Journal of Diabetes Science and Technology, 2011. **5**(4): p. 871-878.
56. Marling, C.R., N.W. Struble, R.C. Bunescu, J.H. Shubrook, and F.L. Schwartz, *A Consensus Perceived Glycemic Variability Metric.* Journal of Diabetes Science and Technology, 2013. **7**(4): p. 871-879.

57. Wiley, M., R. Bunescu, C. Marling, J. Shubrook, and F. Schwartz. *Automatic Detection of Excessive Glycemic Variability for Diabetes Management*. in *2011 10th International Conference on Machine Learning and Applications and Workshops*. 2011. Honolulu, HI, USA.
58. Bertachi, A., L. Biagi, I. Contreras, N. Luo, and J. Vehí, *Prediction of Blood Glucose Levels And Nocturnal Hypoglycemia Using Physiological Models and Artificial Neural Networks*. 2018. p. 85-90.
59. Ngo, C.Q., B.C.Q. Truong, T.W. Jones, and H.T. Nguyen, *Occipital EEG Activity for the Detection of Nocturnal Hypoglycemia.* Conf Proc IEEE Eng Med Biol Soc, 2018. **2018**: p. 3862-3865.
